# Genomic insights into trichome and inflorescence evolution of the palaeopolyploid tree genus *Greyia*

**DOI:** 10.1111/tpj.71134

**Published:** 2026-10-20

**Authors:** Xiao Ma, Jiyang Chang, Jana Botes, Yves Van de Peer, Dave Kenneth Berger

**Affiliations:** ^1^ Department of Plant Biotechnology and Bioinformatics Ghent University 9052 Ghent Belgium; ^2^ VIB Center for Plant Systems Biology 9052 Ghent Belgium; ^3^ Department of Plant and Soil Sciences Forestry and Agricultural Biotechnology Institute (FABI), University of Pretoria Pretoria 0028 South Africa; ^4^ Department of Biochemistry, Genetics and Microbiology University of Pretoria Pretoria 0028 South Africa; ^5^ College of Horticulture, Academy for Advanced Interdisciplinary Studies, Nanjing Agricultural University Nanjing 210095 China

**Keywords:** Geraniales, Francoaceae, *Greyia*, palaeo‐hexaploidisation, flower development, phylogenomics, MBW complex, MADS‐box, *FT*‐*LFY*

## Abstract

The southern African endemic tree *Greyia* Hook. & Harv. (Francoaceae, Geraniales) comprises three species distinguished by striking variation in trichome morphology and inflorescence architecture, yet the genomic and regulatory basis of these traits has remained largely unexplored. Here, we present chromosome‐scale genome assemblies for all recognised *Greyia* species, namely *Greyia radlkoferi*, *Greyia sutherlandii*, and *Greyia flanaganii*, providing the first high‐quality genomic resources for Geraniales. Phylogenomic analyses placed Geraniales as sister to Crossosomatales, with divergence at ~103 million years ago (Mya), and indicated that *Greyia* diversified recently during the Quaternary (~1.39 Mya). Comparative genomics revealed a *Greyia*‐specific palaeo‐hexaploidisation event dated to ~87 Mya, followed by fractionation but with extensive retention of ancestral gene sets in either triplicated or duplicated states across syntenic regions. Transcriptomic analyses revealed regulatory divergence in the conserved MYB–bHLH–WD40 (MBW) network likely associated with variation in trichome morphology, including undetectable *GL1* expression and altered *GL3/EGL3* expression in *G. sutherlandii*. In addition, analyses of flower and inflorescence traits identified expansion of MADS‐box transcription factor families and species‐specific *cis*‐regulatory divergence in *LFY* and *FT*. Notably, predicted AGL6 binding sites are retained in the *LFY* and *FT* promoters of *G. radlkoferi* and *G. sutherlandii* but absent in *G. flanaganii*, consistent with differences in inflorescence density among species. Together, these results demonstrate how polyploidy and regulatory divergence of conserved developmental pathways have shaped morphological diversification in *Greyia*.

## Significance Statement

Understanding how morphological diversity arises from conserved pathways is a central evolutionary challenge, particularly in lineages shaped by ancient polyploidy. We present the first chromosome‐scale genomes for Geraniales, focusing on three southern African endemic *Greyia* species. We demonstrate that regulatory divergence following a lineage‐specific palaeo‐hexaploidisation event, rather than changes in gene content, drives variation in trichome morphology and inflorescence architecture.

## INTRODUCTION

*Greyia* Hook. & Harv. is a small genus of trees/shrubs endemic to South Africa and Eswatini (Dahlgren & van Wyk, 1988), comprising three currently recognised species: *Greyia sutherlandii* Hook. & Harv., *Greyia radlkoferi* Szyszyl., and *Greyia flanaganii* Bolus (Killick & Kimpton, 1977; Phillips & Gower, 1923; Steyn et al., 1999). They are commonly known as bottlebrush or beacon trees on account of the spectacular red inflorescences of *G. sutherlandii* and *G. radlkoferi* that appear in late winter/early spring prior to leaf sprouting in these deciduous species (Figure 1a,b). *Greyia* are prolific nectar producers, thus contributing ecosystem services to a range of generalist bird and insect pollinators at times of nectar scarcity in the mountainous landscapes of the Great Escarpment and Drakensberg of South Africa (Dahlgren & van Wyk, 1988). *Greyia radlkoferi* (woolly bottlebrush) is the most northerly species distributed in Limpopo and Mpumalanga provinces (De la Cruz, 2016), with *G. sutherlandii* (glossy bottlebrush) overlapping in distribution in the latter province, but mainly occupying KwaZulu‐Natal Province (Mbambezeli, 2016). The most southerly species, *G. flanaganii*, is called the Kei bottlebrush on account of its limited distribution along the Kei River and its tributaries in the Eastern Cape Province of South Africa (Mbambezeli, 2002). Unlike its deciduous congeneric species, *G. flanaganii* is an evergreen and lower‐elevation species and possesses distinct inflorescences characterised by sparsely arranged, lax, pendulous, urn‐shaped red flowers (Figure 1c). Differentiation between *G. radlkoferi* and *G. sutherlandii* in the field is often based on the ‘woolly’ abaxial leaf morphology of *G. radlkoferi*, in contrast to the glabrous (hairless) leaves of *G. sutherlandii*. (Figure 1a,b). However, several authors have noted that this can be an unreliable identification trait (Dahlgren & van Wyk, 1988).

**Figure 1:**
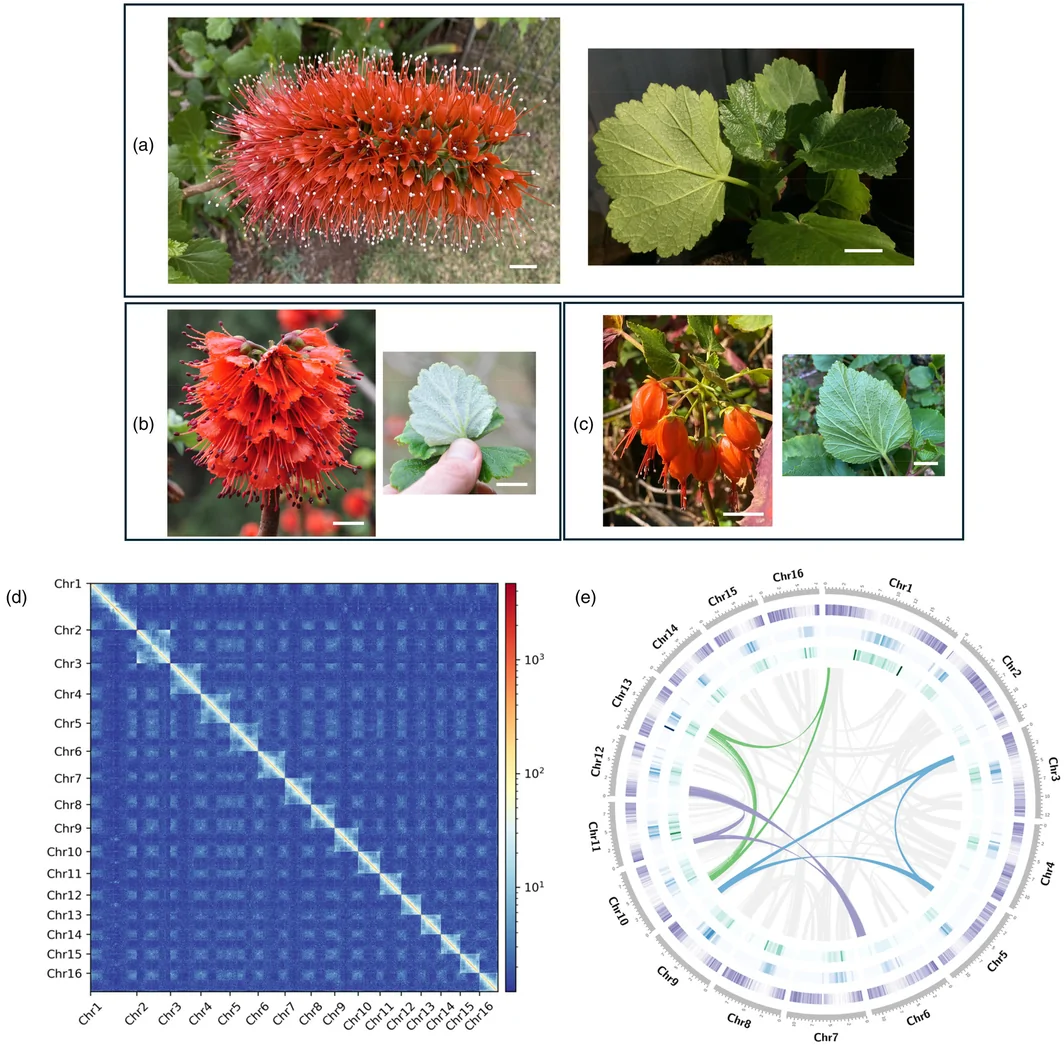
*Greyia* species morphology, genome assembly of *Greyia radlkoferi*. (a–c) Inflorescence and abaxial leaf morphologies of *Greyia sutherlandii*, *G. radlkoferi*, and *Greyia flanaganii*, respectively. Scale bars = 2 cm. (d) Hi‐C interaction heatmap of the assembled *G. radlkoferi* genome. (e) *Greyia radlkoferi* genome assembly. The tracks from the inside to the outside correspond to Copia elements, Gypsy elements, gene density per 100 kb and chromosomes. The innermost grey curves represent all syntenic regions, while the three coloured lines denote representative syntenic regions retained in triplicate.

Apart from the ecological importance of *Greyia* spp., they have economic value due to their horticultural potential as garden plants, having been popular already in the last century in Europe and South Africa (Nonyane, 2010). Extracts from *Greyia* leaves have also attracted interest for medicinal applications, particularly for the treatment of hyperpigmentation, with studies demonstrating anti‐tyrosinase activity in *G. radlkoferi* and *G. flanaganii* without detectable cytotoxic or mutagenic effects (Lall et al., 2016; Mapunya et al., 2011). The active biomarker compound was identified as 2′,4′,6′‐trihydroxydihydrochalcone (Lall et al., 2016). A public‐private partnership is underway in South Africa to develop the technology pipeline from orchard cultivation to herbal remedy formulation for the commercialisation of *Greyia* extracts for skin disorders.

Taxonomic classification of *Greyia* based on morphology has long been problematic. Since its initial placement in Saxifragaceae by Harvey (1862), *Greyia* has subsequently been affiliated with Melianthaceae or treated as a separate family (Greyiaceae), reflecting uncertainty in its systematic position (Dahlgren & van Wyk, 1988). To date, the amount of phylogenetically informative molecular data available for *Greyia* is limited with only plastid gene or ITS data for one or two individuals available on Genbank (Linder et al., 2006; Morgan & Soltis, 1993). Recently, DNA barcoding genes (ITS, *matK*, *psbA‐trnH*, *trnLF*) were sequenced from geographically separated populations of *Greyia* (five trees per species) (Botha, De Canha, et al., 2026). However, phylogenetic analysis indicated there were insufficient polymorphisms to confidently differentiate the species from one another, although *G. flanaganii* was the most distinct. In further work aimed at differentiating the three *Greyia* species, RADseq data from two to three ‘species‐representative’ *Greyia* individuals was used for SNP discovery (Botha, Maduna, et al., 2026). Natural populations sampled across the South African distribution range of *Greyia* could be differentiated into the three species groups using the developed 23‐SNP assay; however, there was admixture in transition zones (Botha, Maduna, et al., 2026). Nevertheless, molecular‐based classification of *Greyia* has placed it in Geraniales, which comprises two families, Geraniaceae and Francoaceae with *Greyia* in the latter (The Angiosperm Phylogeny Group et al., 2016). There are seven genera in Geraniaceae (*California*, *Erodium*, *Geranium*, *Hypseocharis*, *Monsonia*, *Pelargonium*, *Sarcocaulon*), and seven genera in Francoaceae (*Balbisia*, *Bersama*, *Francoa*, *Greyia*, *Melianthus*, *Rhynchotheca*, *Viviania*). Francoaceae takes precedence over its synonyms Greyiaceae and Melianthaceae (The Angiosperm Phylogeny Group et al., 2016). Linder et al. (2006) presented molecular data for members of this family with a study focused on *Melianthus* species with *Greyia* as the outgroup. DNA sequence data for a nuclear (ITS) and plastid (*trnL‐ trnF*, *psbA*‐*trnH*) gene fragments were determined, which confirmed the monophyly of *Melianthus* distinct from *Greyia*.

More broadly, the phylogenetic placement of Geraniales has remained unresolved, largely due to the limited availability of genomic data. Previous phylogenetic studies, summarised in the APG IV classification and based primarily on plastid and nuclear ribosomal DNA sequences, placed Geraniales and Myrtales as early‐diverging sister lineages within the malvid clade. Another phylogenetic analysis based on 4792 chloroplast genomes, representing all currently recognised families, also supported Geraniales and Myrtales as the sister group to the remaining malvids (Li et al., 2021). However, a recent nuclear phylogenomic study using 353 protein‐coding genes of angiosperms (Angiosperms353) (Johnson et al., 2019) recovered a different topology, supporting a sister relationship between Geraniales and Crossosomatales, with Myrtales grouping instead with Zygophyllales (Zuntini et al., 2024). These four orders formed a clade that is sister to rosids, but not to the malvid clade. Although plastid‐ and nuclear‐based phylogenies are largely congruent for most angiosperm orders, Geraniales represents a notable exception. Importantly, *Greyia* species are poorly represented or entirely absent from these large‐scale phylogenomic datasets, including the Angiosperms353 framework. Genome‐scale data from *Greyia* therefore provide a critical opportunity to clarify the phylogenetic position of Geraniales and to establish a much‐needed genomic reference for this understudied order.

The remarkable evolutionary success and diversification of angiosperms are intrinsically linked to genomic plasticity, with whole‐genome duplication (WGD) serving as a pivotal driver of morphological and ecological innovation (Cai et al., 2019; Walden et al., 2020). Following WGD, most genomes experience a phase of reorganisation, including the loss, pseudogenisation, subfunctionalisation and neofunctionalisation of coding sequences (Heslop‐Harrison et al., 2023; Soltis et al., 2016; Wendel, 2015). These processes create opportunities for functional diversification and adaptive radiation (Soltis et al., 2015; Van de Peer et al., 2021). *Greyia* exhibits pronounced variation in trichome morphology and inflorescence architecture (Figure 1a–c), making it an attractive system to investigate if and how WGD‐derived processes contribute to morphological diversification within a restricted lineage. For example, floral ontology and development in Geraniales have been the subject of several morphological studies (Jeiter, Hilger, et al., 2017; Ronse Decraene et al., 2001), including a comparison between *Greyia* spp. and *Francoa* (Ronse Decraene & Smets, 1999). Overall flower morphologies in Geraniales are distinct, since genera such as *Melianthus* and *Pelargonium* have zygomorphic flowers, in contrast to others such as *Greyia* and *Geranium* with actinomorphic flowers (Jeiter, Weigend, & Hilger, 2017; Steyn et al., 1987). However, the absence of annotated genome‐scale resources in Geraniales has so far limited exploration of the molecular basis of these morphological adaptations.

Leaf surface morphology is a characteristic that has been used to differentiate the *Greyia* species as *G. radlkoferi* leaves have abundant hairs (non‐glandular trichomes), especially on the abaxial side (Botha, De Canha, et al., 2026; Steyn, 1974). In contrast, *G. sutherlandii* and *G. flanaganii* leaves are predominantly glabrous (Botha, De Canha, et al., 2026; Steyn, 1974). In addition, microscopy of leaf sections has shown that all three species possess glandular trichomes (Steyn, 1974). Trichome morphology has long been recognised as a valuable taxonomic character in Pelargonium within the Geraniales (Oosthuizen, 1983). However, the developmental genetic mechanisms underlying trichome diversity remain unexplored in Geraniales, including *Greyia*.

Our current knowledge on trichome development is mostly derived from studies of *Arabidopsis thaliana*, which possesses non‐glandular, unicellular branched trichomes. The C2H2 zinc‐finger transcription factor (TF) *GLABROUS INFLORESCENCE STEMS* (*GIS*) and its subfamily members act upstream of the MYB‐bHLH‐WD40 (MBW) transcriptional complex—comprising *GLABRA 1* (*GL1*), *GLABRA 3* (*GL3*)/*ENHANCER OF GLABRA 3* (*EGL3*), and *TRANSPARENT TESTA GLABRA 1* (*TTG1*)—to regulate non‐glandular trichome initiation in Arabidopsis (Grebe, 2012; Oppenheimer et al., 1991; Walker et al., 1999; Zhang et al., 2003). Many eudicots rely on an MBW complex for trichome initiation, similar to *A. thaliana*. Distinct short and long unicellular trichomes on the petals of *Nigella damascena* are initiated through the action of an MBW complex (Peng et al., 2025). *Solanum lycopersicum* (tomato) relies on an MBW complex with competing R2R3‐MYB members to regulate the expression of a repressor of multicellular trichome initiation (Qi et al., 2025). The MBW complex is also involved in the initiation of glandular trichomes in some species, such as *Artemisia annua* (Jiang et al., 2025; Yuan et al., 2025), where upregulation of *AaTTG1* led to increased glandular trichome density and artemisinin content. However, the regulatory networks controlling multicellular trichome development have diverged in some plant lineages. For example, *GL1* orthologues do not appear to regulate trichome development in tobacco, unlike in *A. thaliana* (Payne et al., 1999).

Here, we present high‐quality, chromosome‐scale genome assemblies and annotations for all three recognised *Greyia* species, representing the first annotated genomic resources for Geraniales. Using comparative genomics and transcriptomic analyses, we reconstruct the evolutionary history of *Greyia*, identify a lineage‐specific palaeo‐hexaploidisation event, and examine how gene retention, expression divergence, and *cis*‐regulatory evolution have shaped trichome differentiation and inflorescence architecture. Together, these data provide new insights into the genomic mechanisms underlying morphological diversification in *Greyia* and establish a foundation for future evolutionary and functional studies within Geraniales.

## RESULTS

### Assemblies and annotations of three *Greyia* genomes

Prior to *de novo* assembly, genome sizes were estimated by *k*‐mer analysis of Illumina short reads, indicating genome sizes of 178–192 Mb for *Greyia* (2*n* = 2*x* = 32; Figure S1). Oxford Nanopore long‐read sequencing generated 16.1 Gb (102×), 13.4 Gb (75×), and 15.0 Gb (84×) of data for *G. radlkoferi*, *G. sutherlandii* and *G. flanaganii*, respectively (Table S1a). *De novo* assemblies using Flye v2.9.3 (Kolmogorov et al., 2019) yielded contig‐level assemblies of comparable sizes (175–178 Mb), consistent with *k*‐mer estimates. The *G. radlkoferi* assembly comprised 314 contigs (N50 = 5.9 Mb), *G. sutherlandii* 394 contigs (N50 = 2.5 Mb), and *G. flanaganii* 261 contigs (N50 = 5.7 Mb) (Table 1). To generate chromosome‐scale assemblies, 27.5 Gb (152×) of Hi‐C data were produced for *G. radlkoferi*, enabling scaffolding into 16 pseudomolecules with ~5 Mb remaining unplaced (Figure 1d). Using *G. radlkoferi* as a reference, genome‐guided scaffolding was applied to *G. sutherlandii* and *G. flanaganii* (Figure S2). All three assemblies were resolved into 16 pseudomolecules, anchoring 97.7–98.9% of contigs (173–174 Mb) and achieving scaffold N50 values of 9.9–11.2 Mb (Table 1). Assembly quality was further validated by mapping RNA‐seq reads from multiple tissues, with 96.4–98.6% alignment (Table S1b,c). Benchmarking Universal Single‐Copy Orthologues (BUSCO) analysis using the eudicots_odb10 dataset confirmed high completeness (98.5–98.6%; Table 1). Together, these results demonstrate the high quality of the three *Greyia* genome assemblies.

**Table 1:** Statistics of the three *Greyia* genome assemblies and annotations

Species	*G. radlkoferi*	*G. sutherlandii*	*G. flanaganii*
Assembly features			
Estimated genome size (Mb)^a^	178	/	192
Number of contigs	314	394	261
N50 contig length (Mb)	5.9	2.5	4.7
Total size of contigs (Mb)	175	177	178
Number of pseudo‐chromosomes	16	16	16
Total size of pseudo‐chromosomes (Mb)	173	173	174
N50 scaffold length (Mb)	11.2	9.9	9.9
BUSCO (complete)	98.6%	98.6%	98.5%
TE annotation features			
Repeat content	27.0%	28.5%	29.1%
LTR elements	11.8%	12.2%	11.6%
Copia	4.01%	4.25%	3.89%
Gypsy	5.3%	5.1%	4.7%
DNA transposons	1.6%	1.5%	2.1%
Gene annotation features			
Number of gene models	27 222	26 878	27 861
Mean gene length (bp)	2848	2883	2832
Mean coding sequence length (bp)	1231	1258	1221
Mean exons number per gene	5.39	5.43	5.36
Mean exon length (bp)	228	231	228
Mean intron length (bp)	369	368	370
BUSCO (complete)	99.6%	99.6%	99.3%
Complete and single‐copy	94.2%	93.8%	93.5%
Complete and duplicated	5.3%	5.8%	5.8%
Fragmented	0.1%	0.1%	0.4%
Missing	0.3%	0.3%	0.3%
OMArk (complete)	94.1%	94.1%	94.3%

^a^ Genome size was estimated using *k*‐mer analysis of Illumina reads (Figure S1).

Repetitive elements account for 27.0, 28.5, and 29.1% of the *G. radlkoferi*, *G. sutherlandii* and *G. flanaganii* genomes, respectively. Long terminal repeat retrotransposons (LTR‐RTs) dominate as the major transposable element class, accounting for ~12% of each genome, with Gypsy (~5%) and Copia (~4%) being the most abundant LTR families (Table 1). As observed in other plant genomes, transposable element density is inversely correlated with gene density across the chromosomes (Figure 1e). Following transposable element prediction and masking, protein‐coding genes were annotated through an integrated approach combining evidence‐based and *ab initio* methods (see Methods). The high gene density in these compact genomes led to the frequent merging of adjacent genes in initial computational annotations. We markedly improved genome annotation quality through extensive manual curation (see Methods), with BUSCO completeness increasing from 90 to >99%. The final annotations comprise 27 222 genes in *G. radlkoferi*, 26 878 in *G. sutherlandii*, and 27 861 in *G. flanaganii* with transcriptome support (TPM >1) for 86.7, 82.5, and 80.2% of predicted genes, respectively. BUSCO (v5.7.1) confirmed these as near‐complete genome annotations (99.3–99.6% complete; ≤0.3% missing; eudicots_odb10), with comparable gene structures among species (Table 1; Figure 1f). Independent evaluation with OMArk (v0.3.1) yielded slightly different but consistent quality metrics (Table 1). Functional annotation using eggNOG‐mapper and InterProScan assigned putative roles to 93.1–94.2% of all genes.

### Phylogenomics of Geraniales

Previous phylogenetic frameworks have placed Geraniales either as sister to Myrtales based on plastid data or as sister to Crossosomatales based on nuclear gene analyses (Li et al., 2021; The Angiosperm Phylogeny Group et al., 2016; Zuntini et al., 2024), highlighting uncertainty in the placement of this order within rosids. To clarify the phylogenetic position of Geraniales, we conducted a genome‐based phylogenomic analysis using *Greyia* as a representative. We analysed 17 genomes spanning 14 orders within the superrosids: Brassicales, Malvales, Sapindales, Oxalidales, Malpighiales, Celastrales, Fagales, Rosales, Cucurbitales, Fabales, Geraniales, Crossosomatales, Zygophyllales and Myrtales, along with two monocot outgroups (Table S2). This analysis assigned 520 016 genes (92.4% of 563 009 total) to 43 164 orthogroups, including 401 highly conserved single‐copy genes used for phylogenetic reconstruction. Among the three *Greyia* species analysed, we identified 17 048 conserved orthogroups (89.5%) present in all three species, 1488 orthogroups (7.8%) shared by two species, and 519 species‐specific orthogroups (2.7%), indicating a high degree of genomic conservation.

We reconstructed concatenation‐ and coalescent‐based species trees using 401 single‐copy nuclear genes. The two approaches yielded largely congruent topologies. In both trees, Myrtales occupied an early‐diverging position among the sampled superrosids, and *Greyia* was strongly supported as sister to *Euscaphis japonica*, supporting a close relationship between Geraniales and Crossosomatales (Figure 2a; Figure S3). Comparison with the Angiosperms353‐based topology of Zuntini et al. (2024) revealed agreement in the recovery of a Geraniales‐Crossosomatales clade but disagreement regarding its deeper placement within rosids. Zuntini et al. (2024) recovered Geraniales‐Crossosomatales as the earliest‐diverging rosid lineages, with Myrtales diverging next (Figure 2b). In contrast, our concatenated tree placed Geraniales‐Crossosomatales as sister to a clade comprising fabids and malvids (Figure 2a), whereas the ASTRAL tree placed it as sister to fabids, although with relatively weak support (Figure S3). To assess the gene‐tree discordance underlying this conflict, we examined ASTRAL quartet frequencies for the alternative relationships among Geraniales‐Crossosomatales, fabids, and malvids. The topology grouping Geraniales‐Crossosomatales with fabids was the most frequent in both the nucleotide‐ and amino acid–based analyses (0.37 and 0.38, respectively), followed closely by the topology grouping Geraniales‐Crossosomatales with malvids (Figure S3). The alternative grouping of fabids with malvids received frequencies of 0.29 in both datasets (Figure S3). The relatively even distribution of quartet frequencies indicates substantial discordance among individual gene trees and highlights the persistent uncertainty surrounding the deeper placement of the Geraniales‐Crossosomatales clade.

**Figure 2:**
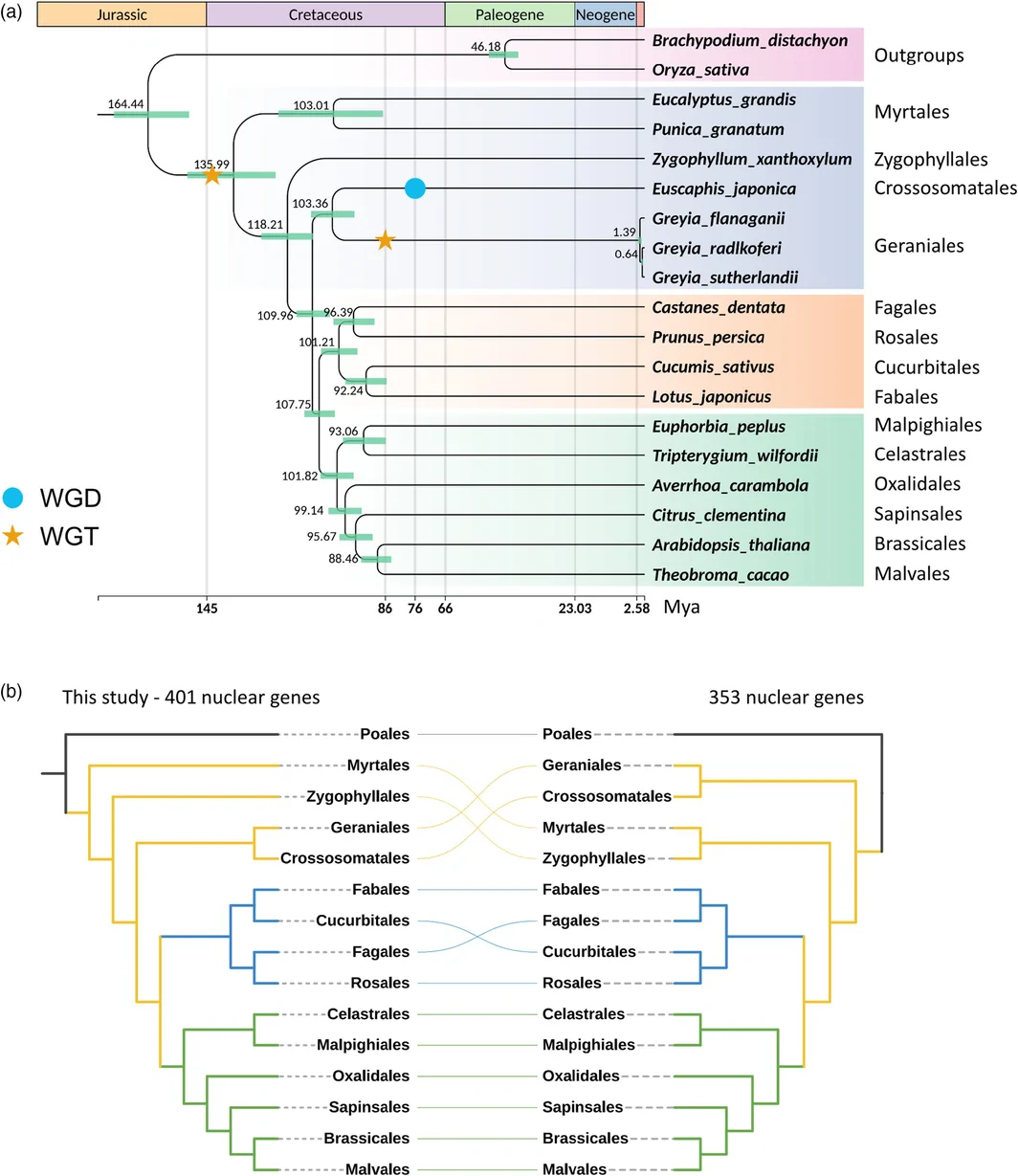
Time‐calibrated phylogeny of *Greyia* and corresponding tanglegram showing topological discrepancies between the current study (left) and a schematic tree based on 353 nuclear genes (Angiosperms353 dataset) published previously (right) (Zuntini et al., 2024). (a) Time‐calibrated tree using 401 single‐copy genes based on genomic data. Only the whole‐genome duplication (WGD) and palaeo‐hexaploidisation events for the groups under study are indicated. (b) Tanglegram at the ordinal level between the tree of the current study (left) and the schematic tree based on 353 nuclear genes (right). In the tanglegram, identical orders are connected across the two trees, enabling visual assessment of topological congruence and incongruence.

We subsequently used the concatenated topology as a phylogenetic framework for divergence‐time estimation, as it was broadly congruent with the ASTRAL tree. The time‐calibrated analysis indicated that Geraniales and Crossosomatales diverged at ~103 Mya (96.05–110.43 Mya; Figure 2a; see Methods). Within *Greyia*, divergence time estimates indicate that *G. flanaganii* represents the earliest split, occurring approximately 1.39 Mya (0.85–2.03 Mya), followed by the split between *G. radlkoferi* and *G. sutherlandii* at 0.64 Mya (0.33–1.01 Mya) (Figure 2a). These results suggest that diversification within *Greyia* took place during the Quaternary period.

To further resolve relationships within Geraniales, we incorporated transcriptomic data from six species with BUSCO completeness greater than 65% (Table S3). This analysis recovered two well‐supported clades corresponding to Geraniaceae and Francoaceae. The Geraniaceae clade included *Pelargonium dichondrifolium*, *Monsonia marlothii*, *California macrophylla* and *Erodium chrysanthum*, whereas the Francoaceae clade comprised *Greyia*, *Melianthus villosus*, and *Francoa sonchifolia* (Figure S4). In Francoaceae, despite their geographic separation, *F. sonchifolia* from South America shows the closest affinity to the South African *Greyia*, with *M. villosus* forming a sister group to the *Francoa*‐*Greyia* lineage (Figure S4). Our findings are consistent with previous phylogenetic analyses based on ITS and *trnL‐F* sequences (Palazzesi et al., 2012; Sytsma et al., 2014), providing additional support for the robustness and stability of evolutionary relationships among genera within Geraniales.

### A *Greyia*‐specific palaeo‐hexaploidisation event

To reconstruct the polyploidisation history of *Greyia*, we applied multi‐method approaches (see Methods). Analysis of synonymous substitution rates (*K*_S_) using syntenic paralogs revealed two clear peaks (~0.52 and ~1.34; Figure 3a) in all *Greyia* species, indicative of two polyploidisation events. The sister lineage *E. japonica* similarly exhibited two *K*_S_ peaks (~0.34 and ~1.21), consistent with previously reported WGD events (Sun et al., 2021). The older *K*_S_ peaks in *Greyia* (~1.34) and *E. japonica* (~1.21) exceed the orthologous divergence with *Vitis vinifera* (~0.89) (Figure 3a), indicating that they correspond to the shared Gamma event in core eudicots (Chanderbali et al., 2022). To determine whether the younger polyploidisation events were shared, we compared orthologous *K*_S_ values between *Greyia* and *E. japonica*. The orthologue *K*_S_ peak between *G. radlkoferi* and *E. japonica* (~0.85) is higher than the lineage‐specific younger peaks (~0.52 in *Greyia* and ~0.34 in *E. japonica*), indicating that these events occurred independently after lineage divergence (Figure 3a). Consistent with this inference, synteny analyses revealed a 3:1 relationship between *Greyia* and *V. vinifera*, and a 3:2 relationship between *Greyia* and *E. japonica* (Figure 3c), supporting a *Greyia*‐specific triplicated genomic structure consistent with palaeo‐hexaploidisation and an *E. japonica*‐specific WGD (Sun et al., 2021). This *Greyia*‐specific palaeo‐hexaploidisation event was also supported by intragenomic collinearity analysis (Figure 1e). Absolute dating (see Methods) estimates this event at ~86.97 (59.86–102.76) Mya (Figure 3b) and the *E. japonica* WGD at ~76.18 (48.99–99.66) Mya (Figure S5). This much older *Greyia*‐specific palaeo‐hexaploidisation contrasts with the recent Quaternary radiation of extant *Greyia* species, predates their diversification, and is therefore best interpreted as a shared ancestral genomic legacy.

**Figure 3:**
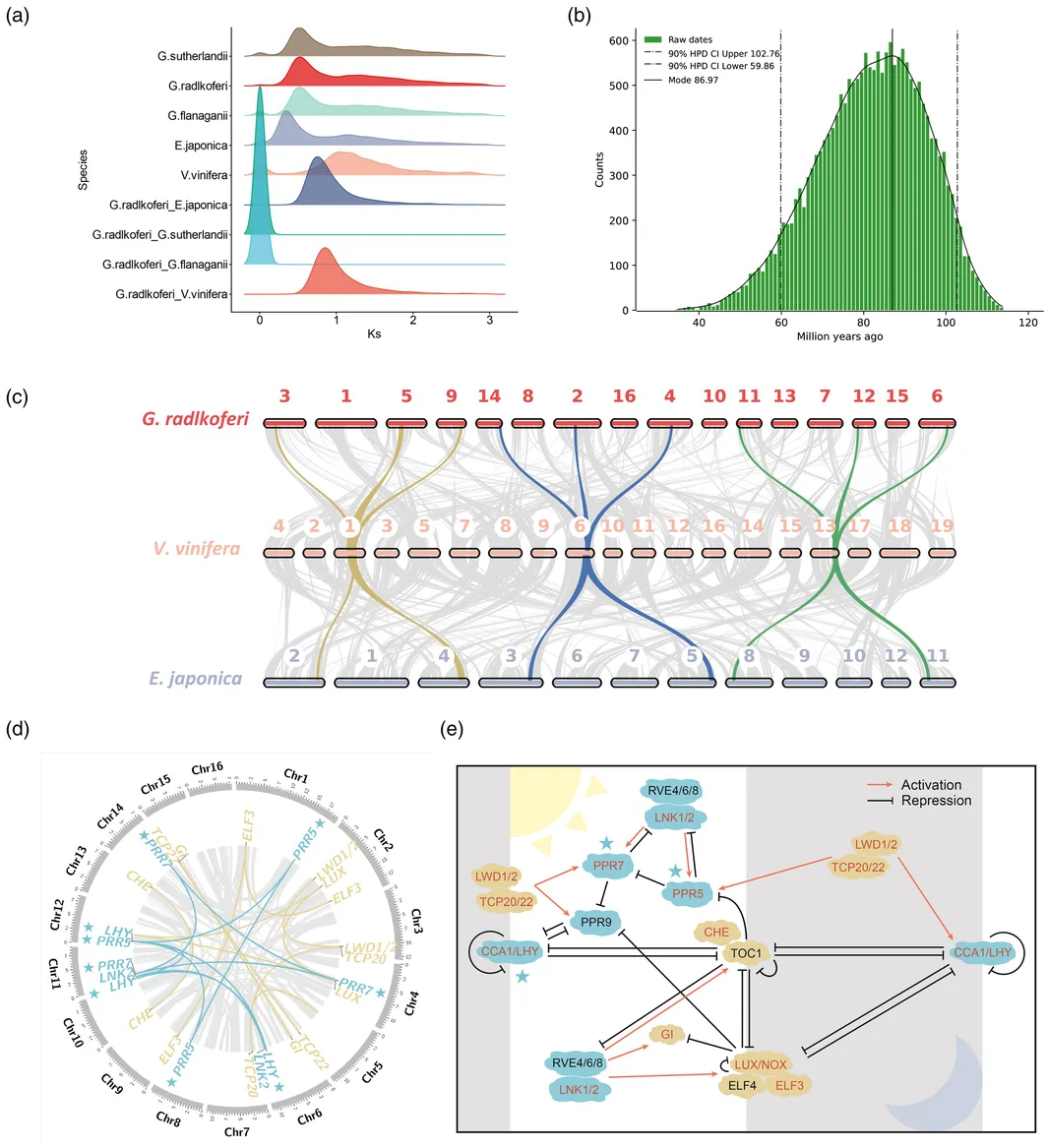
Palaeo‐hexaploidy in *Greyia* species. (a) *K*_S_ distributions for anchor pairs (retained paralogs from the palaeo‐hexaploidisation and whole‐genome duplication [WGD] events) of *Greyia radlkoferi*, *Greyia sutherlandii*, *Greyia flanaganii*, and *Euscaphis japonica* genomes, and for orthologues of *Greyia* and related species. (b) Absolute dating (see Methods) of the *G. radlkoferi* palaeo‐hexaploidisation event. The age distribution was obtained by phylogenomic dating of *G. radlkoferi* paralogs. The solid black line represents the Kernel density estimation (KDE) of the dated paralogs, while the vertical solid black line represents its peak at 86.97 Mya, which was used as the consensus WGD age estimate. The vertical black dotted lines represent the corresponding 90% confidence interval (CI) for the WGD age estimate, 59.86–102.76 Mya. The green histogram shows the raw distribution of dated paralogs. (c) Synteny between *G. radlkoferi*, *Vitis vinifera* and *E. japonica*. The yellow, blue and green lines highlight the 3:1:2 syntenic relationship among these three species. (d) Genes related to the photoperiod transduction pathway adapted from Li et al. (2025) are shown in their syntenic relationships derived from the palaeo‐hexaploidisation event. Blue lines represent transcription factors; yellow lines represent clock proteins. The blue star represents the genes present as triplicates. Duplicated genes include the transcriptional repressor *CCA1 HIKING EXPEDITION* (*CHE*), clock‐associated factors *LIGHT‐INDUCIBLE AND CLOCK‐REGULATED GENE 2* (*LNK2*), *LIGHT‐REGULATED WD 1* (*LWD1*) and *TCP‐DOMAIN FAMILY PROTEIN 20/22* (*TCP20/22*), the evening complex gene *LUX*, and *GIGANTEA* (*GI*); Triplicated genes include *PSEUDO‐RESPONSE REGULATOR 5* (*PRR5*), *PSEUDO‐RESPONSE REGULATOR 7* (*PRR7*), *CIRCADIAN CLOCK ASSOCIATED 1/LATE ELONGATED HYPOCOTYL 1* (*CCA1/LHY1*), a blue light photoreceptor *CRYPTOCHROME 1* (*CRY1*) and a repressor of flowering *EARLY BOLTING IN SHORT DAYS* (*EBS*). (e) The core photoperiod transduction pathway adapted from Li et al. (2025). Genes highlighted in red are retained from the palaeo‐hexaploidisation event. The blue stars indicate genes retained as triplicates, while the remaining red‐highlighted genes are retained as duplicates.

Macrosynteny analysis revealed extensive structural conservation among the three *Greyia* genomes with few large‐scale rearrangements (Figure S6). Approximately 25 000 collinear genes were identified in each pairwise comparison and only three inversions were detected (Figure S7). Intragenomic synteny analyses further showed that ~40% of genes (~11 000 genes) are retained within ~500 well‐conserved duplicated blocks (Table S4). Among these retained duplicated genes (RDGs), approximately 52% derive from the *Greyia*‐specific palaeo‐hexaploidisation event, representing ~3000 gene families per species and a conserved core of 2518 families shared by all three species (Table S4; Figure S8). These shared RDGs are significantly enriched (*P* < 0.05) for functions related to organophosphate and thioester biosynthesis, signal transduction, stomatal movement and pyruvate metabolism (Figure S8).

In addition to duplicated regions, several genomic regions remain preserved in three copies, notably in Chromosome 1 (syntenic with Chr 10/13), Chromosome 3 (Chr 5/9) and Chromosome 6 (Chr 11/12) (Figure 1e). These regions comprise 1080 genes (360 retained triplicated genes, RTGs) conserved across all three species. RTGs are significantly enriched (*P* < 0.05) for functions associated with protein metabolism regulation, circadian activity, rhythmic processes and long‐day photoperiodism (Figure S9). Nearly all copies of the core components involved in the photoperiod pathway (Li et al., 2025) were located in the triplicated blocks retained following the *Greyia*‐specific palaeo‐hexaploidisation (Figure 3d,e). Notably, the core circadian clock transcription factors (*PRR5*, *PRR7*, *CCA1/LHY1*, *CRY1* and *EBS*) are retained as triplicates, whereas several clock‐associated proteins are present as duplicated copies (Figure 3d,e). Expression profiling in *G. radlkoferi* revealed that most RDGs and RTGs display tissue‐biased expression patterns, whereas a subset of genes, including multiple *PRR7* and *GI* copies, show broad expression across flowers, leaves and stem tissues (Figure S10).

### Trichome development

The species‐representative *Greyia* individuals in this study exhibited the characteristic differences in leaf epidermal morphology. *Greyia radlkoferi* leaves were densely covered with trichomes (Figure 1b), whereas *G. sutherlandii* and *G. flanaganii* had glabrous leaves (Figure 1a,c). Our comparative analysis focused primarily on *G. radlkoferi* and *G. sutherlandii*, as stem tissue RNA data were not available for *G. flanaganii* (Table S1b). High‐resolution anatomical scans showed that *G. radlkoferi* leaves were densely covered with long branched non‐glandular trichomes (hairs), whereas *G. sutherlandii* leaves were nearly glabrous (Figure 4a,b). In addition, light microscopy of leaf cross sections showed both *G. radlkoferi* and *G. sutherlandii* also had short glandular trichomes (Figure 4c). These were made up of an epidermal mother cell, a single‐celled stalk and a bulbous secretary head cell that stained intensely with Safranin‐O (Figure 4c), most likely on account of this basic reagent binding to anionic compounds such as acidic cell wall components and nucleic acids of these metabolically active secretory cells (Graham & Trentham, 1998). Only the basal epidermal cell of each long branched trichome was readily visible in the cross sections of *G. radlkoferi* (Figure 4c), as they were torn off during sectioning. These observations are consistent with a previous report describing multicellular uniseriate branched hairs and glands in *G. radlkoferi*, whereas *G*. *sutherlandii* leaves predominantly bore glandular trichomes (Steyn, 1974). However, leaf hairiness of young *G. sutherlandii* leaves can vary even in an individual plant, suggesting differences in trichome density between the two species may be the result of differences in gene expression.

**Figure 4:**
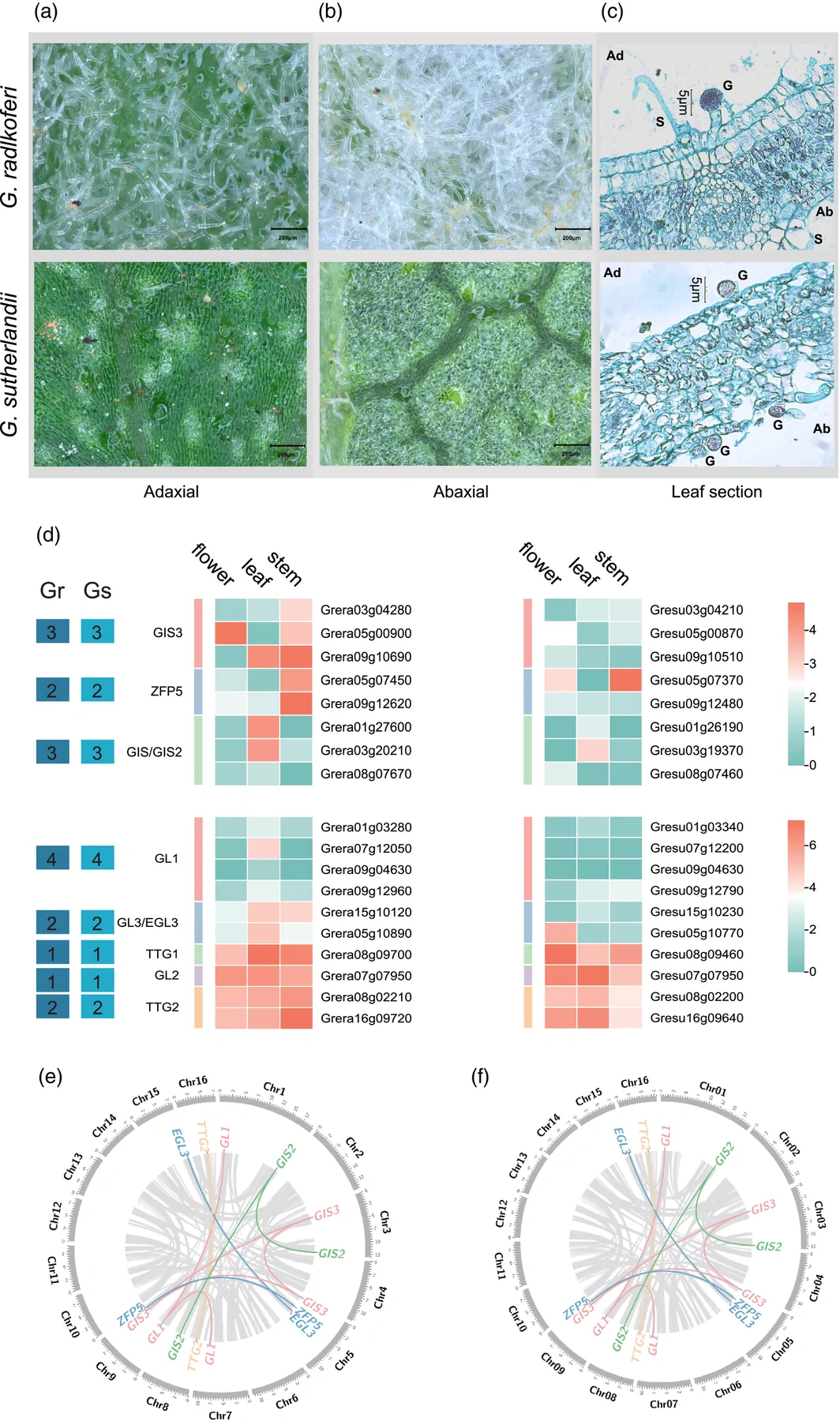
Leaf trichome morphology and the MYB–bHLH–WD40 (MBW) complex in *Greyia*. (a) High‐resolution anatomical scans of the adaxial leaf surface of *Greyia radlkoferi* (left) and *Greyia sutherlandii* (right). Scale bars = 200 μm. (b) High‐resolution anatomical scans of the abaxial leaf surface of *G. radlkofer*i (left) and *G. sutherlandii* (right). Scale bars = 200 μm. (c) Light microscopy of leaf cross sections of *G. radlkoferi* (left) and *G. sutherlandii* (right). Scale bars = 5 μm. Ab, Abaxial; Ad, adaxial; G, glandular trichome; S, simple non‐glandular trichome (partial). (d) Gene copy number and expression patterns of upstream MBW regulators (*GIS3*, *ZFP5*, *GIS*, *GIS2*), the core MBW complex (*GL1‐GL3/EGL3‐TTG1*) and downstream targets (*GL2*, *TTG2*) in *G. radlkoferi* (Gr) and *G. sutherlandii* (Gs). Expression values are scaled by log2 (TPM + 1). TPM is transcripts per million. In the expression profile, the prefix of Grera represents genes in *G. radlkoferi*; Gresu represents genes in *G. sutherlandii*. (e) Genes related to trichome development are shown in their syntenic relationships derived from the palaeo‐hexaploidisation event in *G. radlkoferi*. Lines with different colours represent different gene families. (f) Genes related to trichome development are shown in their syntenic relationships derived from the palaeo‐hexaploidisation event in *G. sutherlandii*. Lines with different colours represent different gene families.

To investigate regulatory differences potentially associated with divergent trichome phenotypes, we examined the MYB–bHLH–WD40 (MBW) complex, the best‐characterised regulatory network controlling non‐glandular trichome initiation in Arabidopsis, as a comparative framework for identifying candidate regulators in *Greyia*. We first identified 311 and 304 MYB TFs and 39 and 38 bHLH TFs in *G. radlkoferi* and *G. sutherlandii*, respectively. We then screened for these TFs with higher expression in leaves relative to flower and stem. Comparative transcriptomic analysis highlighted 13 MYB genes with strong leaf‐specific expression in *G. radlkoferi*, including a homologue of *GL1* (Figure S11). Although the *GL1* orthogroup is represented by four copies in *Greyia*, only a single copy shows high expression in *G. radlkoferi* leaves (Figure 4d). In contrast, the corresponding *GL1* homologue in *G. sutherlandii* exhibited no detectable expression in any sampled tissue (Figure 4d), paralleling the absence of non‐glandular trichomes in this species (Figure 4d). Analysis of the bHLH family further identified two *GL3/EGL3* homologues in *Greyia*. In *G. radlkoferi*, both copies are highly expressed in leaves, whereas in *G. sutherlandii*, one copy showed low expression across all sampled tissues and the other was predominantly expressed in flowers (Figure 4d), indicating marked tissue‐specific divergence. We next examined the broader MBW regulatory network, including upstream regulators (*GIS3*, *ZFP5*, *GIS*, *GIS2*) and downstream targets (*GL2*, *TTG2*). All three *Greyia* species retain identical gene complements for these components (Figure 4c,d). However, expression patterns differed substantially among species. Notably, one copy of *GIS3* showed high expression in *G. radlkoferi* leaves but low expression in *G. sutherlandii* leaves (Figure 4c), closely mirroring the expression pattern of *GL1*. Reduced expression of *GIS/GIS2* was also observed in *G. sutherlandii* leaves (Figure 4c). These observations suggest that although the MBW complex and its regulatory genes are structurally conserved in *Greyia*, divergence in their expression likely underlies the species‐specific variation in trichome development.

To explore potential regulatory mechanisms underlying these expression differences, we analysed DNA methylation profiles from ONT reads. In *G. sutherlandii*, CpG methylation was detected in promoter regions upstream of *GL1* and *GIS3*, whereas no such methylation was detected in the corresponding promoters in *G. radlkoferi* (Tables S5 and S6). These methylation patterns are consistent with the observed transcriptional repression of trichome‐related genes in *G. sutherlandii* and with previously reported variation in leaf hairiness within this species.

Finally, we found several candidate trichome regulators, including *GL1*, *EGL3*, *GIS3*, and *GIS/GIS2*, are present as multiple copies retained after the *Greyia*‐specific palaeo‐hexaploidisation in both *Greyia* species (Figure 4e,f). These paralogs show divergent expression patterns across tissues, with some copies preferentially expressed in floral or vegetative tissues (Figure 4d). Such expression divergence is consistent with possible regulatory partitioning among paralogs following palaeo‐hexaploidisation, although functional analyses are required to determine whether they have undergone subfunctionalization.

### MADS‐box gene families with potential relevance to *Greyia* flower development

*Greyia* species produce conspicuous red flowers that are a prominent feature of the genus (Figure 1a–c). In all three species, flowers bear exserted stamens with red anthers prior to opening (Figure 1b). To investigate the genetic basis underlying floral development in *Greyia*, we focused on MADS‐box TFs, which play central roles in regulating floral organ identity in angiosperms (O'Maoileidigh et al., 2014; Soltis et al., 2007). Among them, type II MADS‐box genes are well‐known for their conserved functions in floral organ specification and distinct expression patterns (Becker & Theissen, 2003; Smaczniak et al., 2012). Genome‐wide analysis identified 87, 89 and 88 putative MADS‐box genes in *G. radlkoferi*, *G. sutherlandii*, and *G. flanaganii*, respectively. All three species shared an identical complement of 47 type II MADS‐box genes (Table S7)—an expansion compared to *A. thaliana*, including homologous genes for the ABCE model of floral organ identities: five *APETALA1*s (*AP1*s) (A function for sepals and petals), two *APETALA3*s (*AP3*s) and two *PISTILLATA*s (*PI*s) (B function for petals and stamen), four *AGAMOUS*s (*AG*s) (C function for stamen and carpel) and six *SEPALLATA*s (*SEP*s) (E function for interacting with ABC function proteins) (Figure 5a). Collinearity analyses indicated that the expansion of type II MADS‐box genes in *Greyia* resulted largely from the palaeo‐hexaploidisation event, including *AP1*, *SEP*, *SVP*, *AGL6* and *SOC1* (Figure 5b–d). For instance, *SEP‐1*, *SEP‐2* and *SEP‐3* are retained as triplicates, whereas *SEP‐4* and *SEP‐5* present as duplicates (Figure 5b–d,g–i). In addition to palaeo‐hexaploidisation‐derived duplications, tandem duplication has also contributed to MADS‐box family expansion, as illustrated by paired *SEP* and *AP1* clusters. *SEP‐4* (Grera02g02950) and *AP1‐4* (Grera02g02960) originated from a tandem duplication on chromosome 2, which is syntenic with another tandem duplication on chromosome 7—*SEP‐5* (Grera07g01690) and *AP1‐5* (Grera07g01680) (Figure 5b–d). Sequence alignment showed only 52% identity between *SEP‐4* and *AP1‐4*, compared with 84% for *SEP‐4*–*SEP‐5* and 76% for *AP1‐4*–*AP1‐5* identity (Figure 5e), supporting tandem duplication predating the palaeo‐hexaploidisation (Figure 5f). Interestingly, the tandem‐derived clusters exhibit distinct expression profiles: *SEP‐4* and *SEP‐5* are highly expressed in flowers, *AP1‐4* is expressed in flowers and stem, and *AP1‐5* in flowers and leaves (Figure 5g–i). These results indicate that tandem duplication and palaeo‐hexaploidisation together contributed to the expanded repertoire of MADS‐box genes in *Greyia*.

**Figure 5:**
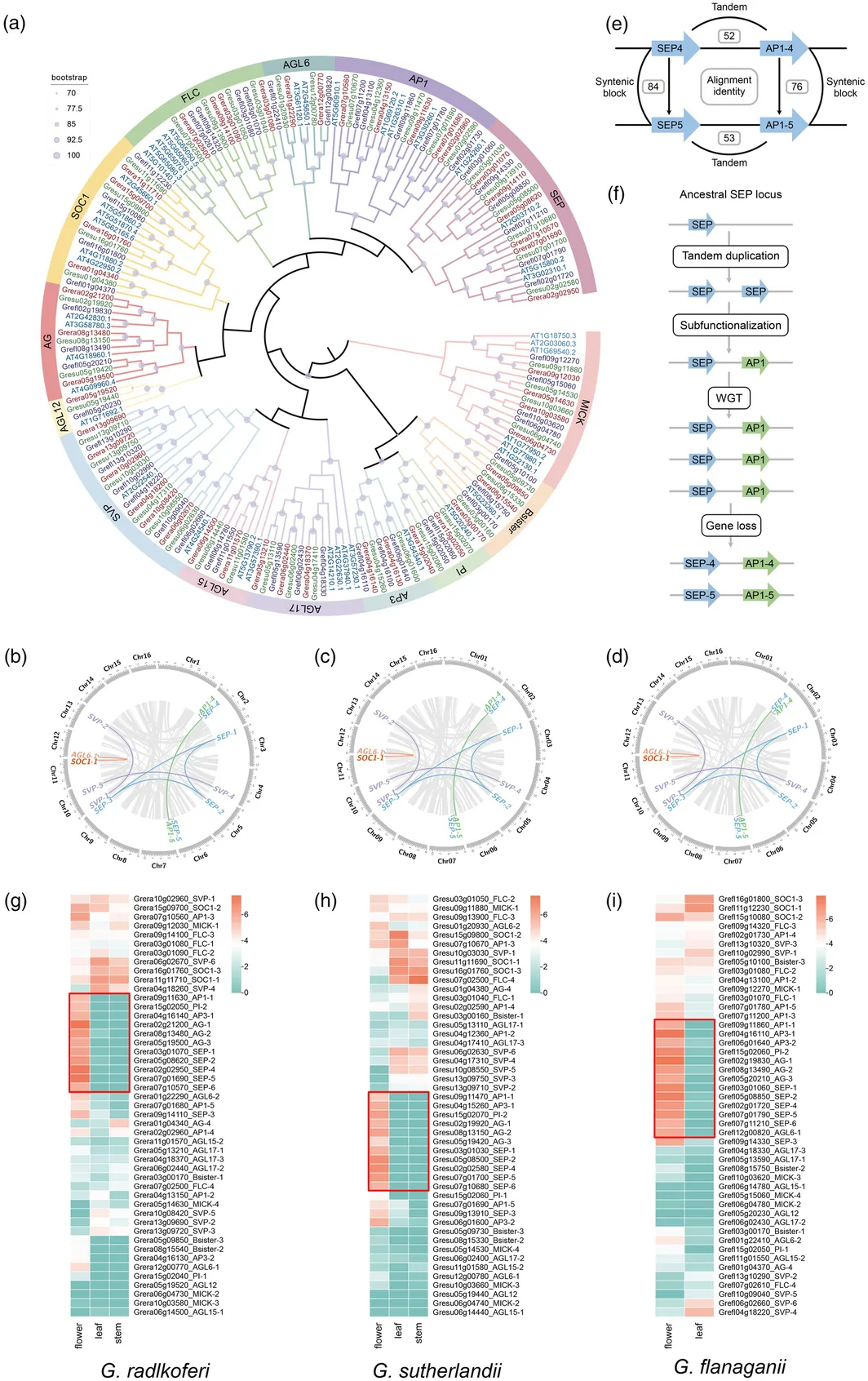
MADS‐box genes in *Greyia* species. (a) The phylogenetic tree of type II MADS‐box genes in three *Greyia* and *Arabidopsis thaliana*. (b–d) The syntenic relationships retained from the palaeo‐hexaploidisation event. Lines with different colours represent different gene families. (e) Sequence alignment identity between two gene clusters (*SEP*‐4 *AP1‐4* and *SEP‐5 AP1‐5*). (f) Reconstruction of the origin of gene clusters of *SEP* and *AP1* (see Text for details). (g–i) Gene expression patterns of type II MADS‐box genes from various organs in the three *Greyia* species. The expression values are scaled by log2 (TPM + 1). TPM is transcripts per million. The red box highlights genes that are highly expressed in flowers, indicating the ABCE homologues in *Greyia*.

We next examined the expression profiles of type II MADS‐box genes across flower, leaf and stem tissues in *Greyia* to identify candidate regulators associated with floral development. Among the five *AP1* paralogs, only *AP1‐1*, the homologue of *APETALA1*, showed consistent, flower‐predominant expression in all three species (Figure 5a,g–i), supporting its conserved association with floral tissues. For B‐class genes, both *PI* and *AP3* have two homologues each, with *PI‐2* and *AP3‐1* showing high expression in flowers, suggesting that they likely cooperate to perform the B function (Figure 5a,g–i). In addition, three C‐function *AG* homologues and five E‐function *SEP* homologues exhibited predominant floral expression, collectively contributing to floral organ identity (Figure 5a,g–i). The expanded set of *AG* homologues in *Greyia* spp. contrasts with many angiosperms that retain only one or two highly expressed copies, suggesting an increased regulatory capacity of the C‐class floral organ identity network (Chang et al., 2023; Ma et al., 2024; Zhang et al., 2020). Together, these expression profiles identify *AP1‐1*, *PI‐2*, *AP3‐1*, three *AG* homologues and five *SEP* homologues as the principal candidate regulators of floral organ identity in *Greyia*.

Within this conserved framework, we further examined *AGL6*, a MADS‐box subfamily closely related to *AP1* and *SEP*. In *Greyia*, two *AGL6* paralogs were identified in all three species. *AGL6‐1* exhibited flower‐specific expression, whereas *AGL6‐2* showed broader expression across flower, leaf, and stem tissues (Figure 5g–i). Quantitative differences of *AGL6‐1* floral expression were observed among species. *AGL6‐1* showed lower floral expression compared to *AP1‐1* in *G. radlkoferi* and *G. sutherlandii*; however, in *G. flanaganii*, *AGL6‐1* is highly co‐expressed with *AP1‐1* in flowers (Figure 5g–i). Phylogenetically, *AGL6* is closely related to *AP1*, and together with *SEP*, these subfamilies form a monophyletic superclade, indicating a common ancestral origin predating the angiosperm/gymnosperm divergence (Becker & Theissen, 2003; Morel et al., 2019). Although the precise relationships among the subfamilies are not fully resolved, our analysis places *SEP* and *AP1* as sister groups, with *AGL6* sister to this clade (Figure 5a).

### Inflorescence development

*Greyia* species are characterised by sympodial branching of shoots with inflorescences forming at the apex but a single side branch developing, causing a layered architecture (Steyn et al., 1987). The inflorescences are unbranched, indeterminate racemes bearing conspicuous red, nectariferous flowers (Figure 1a–c). While *G. radlkoferi*, *G. sutherlandii* and *G. flanaganii* share a similar raceme structure, they differ markedly in flower density. *Greyia radlkoferi* and *G. sutherlandii* produce densely packed racemes with 30–120 flowers and short pedicels (<1.2 cm) (Figure 6a, image 5–8), whereas *G. flanaganii* bears sparser racemes typically with fewer than 12 flowers and conspicuously greater inter‐floral spacing (1.5–3 cm) (Figure 6a, images 1–4). At the level of overall inflorescence organisation, *Greyia* racemes resemble the simple, unbranched, indeterminate raceme of *A. thaliana*. However, because detailed developmental anatomical data for *Greyia* inflorescences are currently limited, we do not assume direct anatomical equivalence between the two systems. Instead, we used conserved flowering and meristem‐identity regulators characterised in Arabidopsis, including *TERMINAL FLOWER 1* (*TFL1*), *LEAFY* (*LFY*), and *FLOWERING LOCUS T* (*FT*), as a comparative framework to identify candidate genes associated with inflorescence variation in *Greyia* (Andres et al., 2015).

**Figure 6:**
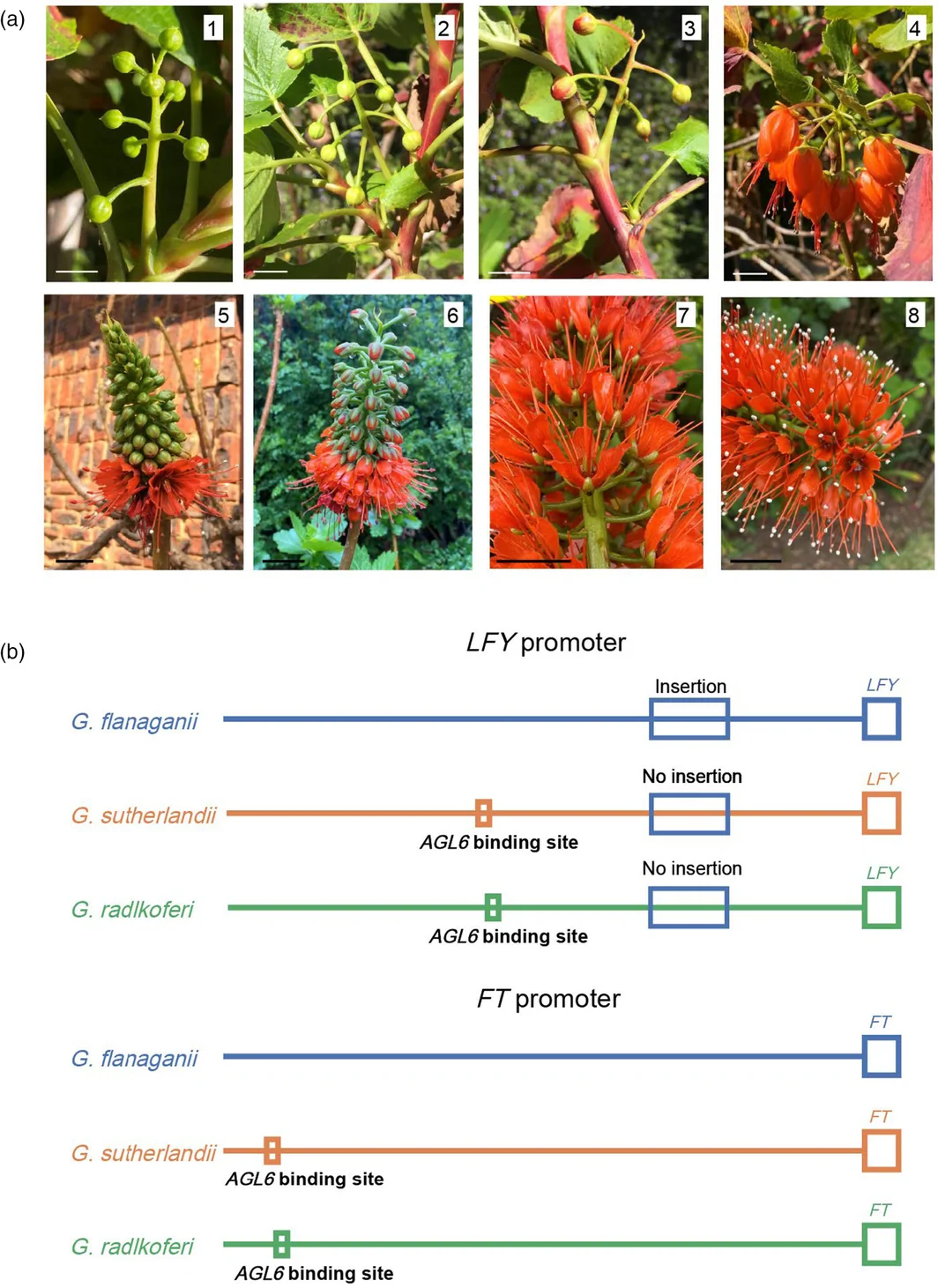
Potential regulation of inflorescence genes in *Greyia*. (a) Inflorescence development from bud to anthesis in *Greyia flanaganii* and *Greyia sutherlandii*. 1–3: *Greyia flanaganii* bud development, illustrating the long pedicels (1.5–3 cm). 4: *Greyia flanaganii* inflorescence at anthesis, illustrating the typical number of flowers per inflorescence (<12), and the pendulous urceolate flower structure with unfused petals in imbricate formation. 5–6: *Greyia sutherlandii* acropetal bud development, illustrating the many flowers per inflorescence (30–120). 7–8: *Greyia sutherlandii* at anthesis, with short erect pedicels (<1.2 cm), and valvate flowers. The panels represent stages 11–13 of flower development as defined for the model plant Arabidopsis in Alvarez‐Buylla et al. (2010). *Greyia radlkoferi* is not shown since its inflorescence development is similar to *G. sutherlandii*. White scale bars = 1 cm; black scale bars = 2 cm. (b) *Cis*‐regulatory divergence of *LFY* and *FT* among *Greyia* species. Promoter lengths are scaled based on sequence alignments. For *LFY*, the promoter spans 2 kb upstream of the translation start site (ATG) in *G. flanaganii*, but is shorter in *G. sutherlandii* (1769 bp) and *G. radlkoferi* (1772 bp) due to the absence of a lineage‐specific insertion. In *G. flanaganii*, this insertion is located 341–566 bp upstream of the *LFY* start codon. Predicted AGL6 binding sites are present in the *LFY* promoters of *G. sutherlandii* (−1026 to −1044 bp) and *G. radlkoferi* (−1030 to −1048 bp), based on motif similarity (*P*‐value = 9.92e‐06; *q*‐value = 0.0237), but are absent in *G. flanaganii*. For *FT*, predicted AGL6 binding sites were detected in the *FT* promoters of *G. sutherlandii* (−1826 to −1844 bp) and *G. radlkoferi* (−1828 to −1846 bp), based on motif similarity (*P*‐value = 2.09e‐06; *q*‐value = 0.00684), but not in *G. flanaganii*.

To investigate whether similar regulators underlie inflorescence development in *Greyia*, we analysed *LFY*, *TFL1* and *FT* across species. Comparative analyses revealed that all three genes display highly conserved coding sequences and gene structures (Figure S12). However, *LFY* and *FT* show clear species‐specific divergence at the promoter level, whereas no such divergence was detected for *TFL1*, indicating regulatory rather than structural variation. *LFY* is retained as a single‐copy gene and exhibits extensive regulatory potential at the promoter level. Promoter analyses of *LFY* (see Methods) predicted 58 putative TF binding sites representing 30 TFs in *G. flanaganii*, compared with 53 sites for 26 TFs in *G. radlkoferi* and 46 sites for 25 TFs in *G. sutherlandii* (Tables S8–S10). Multiple sequence alignments identified a 226‐bp insertion in the *G. flanaganii LFY* promoter, which is absent in *G. radlkoferi* and *G. sutherlandii* (Figure 6b; Figure S13). Motif prediction using the JASPAR 2026 database (Ovek Baydar et al., 2025) indicated that this insertion harbours a putative *PHL12* binding site (Table S8), although the functional relevance of this motif remains unclear, as *PHL12* has not previously been implicated in flowering regulation. Moreover, a predicted AGL6 binding site was detected in the *LFY* promoters of *G. radlkoferi* and *G. sutherlandii* but was absent in *G. flanaganii* (Figure 6b; Tables S8–S10). Despite these *cis*‐regulatory differences, *LFY* transcript abundance was extremely low across all sampled tissues, consistent with its restriction to meristematic domains and suggesting that *LFY* activity at early inflorescence stages is not captured by the present RNA‐seq dataset. Although direct binding of AGL6 to the *LFY* promoter has not been experimentally demonstrated, the known role of AGL6 in promoting floral transition through activation of *FT* and repression of *FLC/FLM* suggests a potential contribution to species‐specific modulation of *LFY* activity in *Greyia* (Kong et al., 2022; Yoo et al., 2011; Yu et al., 2017).

Each *Greyia* genome contains two *FT* paralogs from the *Greyia*‐specific palaeo‐hexaploidisation event; however, only one copy showed detectable expression in the sampled tissues and was therefore retained for further analysis, consistent with functional divergence, as reported for *FT* paralogs in poplar (Bohlenius et al., 2006). Promoter analysis of this expressed *FT* copy revealed 73 predicted TF binding sites corresponding to 18 TFs in *G. flanaganii*, and 86 sites representing 26 TFs in both *G. radlkoferi* and *G. sutherlandii* (Tables S11–S13). Similar to *LFY*, a predicted AGL6 binding site was detected in the *FT* promoters of *G. radlkoferi* and *G. sutherlandii* but was absent in *G. flanaganii* (Figure 6b; Tables S11–S13), indicating species‐specific divergence in the *cis*‐regulatory landscape. Expression profiling showed that in the deciduous species *G. radlkoferi* and *G. sutherlandii*, *FT* transcripts were predominantly detected in mature flowers at anthesis, whereas no expression was observed in their leaf samples. By contrast, the evergreen species *G. flanaganii* exhibited *FT* expression in both flower and leaf tissues (Figure 6; Figure S14). These spatial expression patterns are consistent with differences in growth habit and phenology, with evergreen species retaining leaves during flowering and deciduous species flowering prior to leaf flush, as described for other woody perennials (Bohlenius et al., 2006; Hsu et al., 2011; Wilkie et al., 2008). The detection of *FT* expression in mature floral tissues is also noteworthy, as transcriptomic studies in Arabidopsis typically report very low *FT* expression in flowers, reflecting its primary role during early floral transition rather than late floral development (Liu et al., 2013; Zeevaart, 2008). In *Greyia*, floral *FT* expression coincides with elevated floral expression of *AGL6‐2* and the presence of predicted AGL6 binding sites in *G. radlkoferi* and *G. sutherlandii*, compared to *G. flanaganii* (Figure S15). Together, these observations highlight coordinated expression and *cis*‐regulatory features associated with floral tissues, but do not by themselves establish a direct regulatory relationship. Clarifying the regulatory relationships among *AGL6*, *FT*, and *LFY*, and evaluating their potential contribution to variation in inflorescence architecture, will require transcriptomic and spatial expression analyses encompassing shoot apical meristems, early floral transition stages and multiple seasonal time points.

## DISCUSSION

*Greyia* is a small genus comprising three species, and the three chromosome‐scale genomes reported here constitute an important genomic resource for elucidating the molecular mechanisms underlying trichome, floral and inflorescence differentiation. Representing all currently recognised species—*G. radlkoferi*, *G. sutherlandii*, and *G. flanaganii*—these assemblies provide the first high‐quality reference genomes for Geraniales and establish a valuable foundation for comparative genomics and evolutionary studies.

*Greyia* species possess relatively small genomes (~180 Mb; Table 1) with low heterozygosity, posing little challenge for genome assembly. However, achieving a high‐quality assembly does not automatically guarantee accurate annotation (Wu et al., 2024). For instance, while BUSCO assessments of recently published telomere‐to‐telomere (T2T) and gapless genomes often achieve impressive completeness rates of ~98%, the evaluation of predicted genes typically falls slightly below this benchmark (Detcharoen et al., 2023; Nie et al., 2023; Xia et al., 2023). Indeed, the compact genome structure of *Greyia* genomes frequently led to adjacent genes being linked together. Here, we took advantage of comprehensive gene predictions and extensive manual curations of gene models (see Methods), achieving a BUSCO completeness score exceeding 99.3%, and over 80% of genes that were supported by transcriptomic evidence—an improvement over many previously reported plant genomes.

The phylogenetic placement of Geraniales has long remained uncertain, largely due to the limited availability of genomic resources from key lineages. Our analyses provide additional genomic evidence for a sister relationship between Geraniales and Crossosomatales, supporting their recognition as a well‐supported clade within superrosids. This relationship is consistent with previous phylogenomic studies, including analyses of the *E. japonica* genome and the large‐scale Angiosperms353 dataset (Sun et al., 2021; Zuntini et al., 2024). Nevertheless, the deeper placement of this clade remains uncertain. Its placement differed between our concatenated and ASTRAL analyses and also from that recovered by Zuntini et al. (2024). Quartet‐frequency analyses further revealed that the three alternative relationships among Geraniales‐Crossosomatales, fabids and malvids occurred at broadly similar frequencies, indicating substantial gene‐tree discordance at this node. This pattern is consistent with rapid successive divergences and associated incomplete lineage sorting during the early diversification of superrosids (Mirarab et al., 2014; Sun et al., 2021). However, incomplete lineage sorting should not be regarded as the only possible explanation, because limited taxon sampling, introgression, gene‐tree estimation error and model misspecification may also contribute to discordance among ancient, short branches. Thus, although the monophyly of the Geraniales‐Crossosomatales clade is well supported, its relationship to the other major superrosid lineages remains unresolved. Additional topological differences were observed within fabids. The relationships among Cucurbitales, Rosales, Fagales and Fabales recovered in our analyses differed from those reported by Zuntini et al. (2024). Although our dataset shares only a limited number of loci with the Angiosperms353 marker set, differences in locus selection alone are unlikely to explain this discrepancy. Kates et al. (2024) analysed a largely independent set of nuclear genes but recovered the same Cucurbitales‐Rosales and Fagales‐Fabales relationships as Zuntini et al. (2024), suggesting that these relationships are comparatively stable. Our more restricted taxonomic sampling in our study, particularly within the major fabid lineages, may instead have contributed to the alternative topology recovered here. Resolving these deep relationships will therefore require the integration of additional genomic resources and the application of complementary phylogenomic frameworks (Chen et al., 2019; Huang et al., 2025; Sanderson et al., 2023; Stull et al., 2020).

Within Geraniales, the combined genomic and transcriptomic evidence supports two major families, consistent with the previous result (Palazzesi et al., 2012). Within Francoaceae, the closest relationship between *Greyia* (South Africa) and *Francoa* (South America) points to a historical biogeographic connection between these lineages across Gondwana. This pattern may reflect either ancient geographic separation following the breakup of Gondwana, with descendants persisting on different continents, or from later long‐distance dispersal across the South Atlantic, followed by diversification in isolation. Divergence dating further indicates that *Greyia* diversified relatively recently during the Quaternary (≤1.4 Mya), likely influenced by climatic fluctuations that promoted ecological specialisation across southern Africa (Fletcher & Thomas, 2010; Paillard, 2015).

Polyploidisation plays a crucial role in plant genome evolution and adaptation to environmental change (Van de Peer et al., 2017). Using multiple complementary approaches, we identified a *Greyia*‐specific palaeo‐hexaploidisation event dated to ~86.97 Mya, in addition to the core‐eudicot Gamma event. This palaeo‐hexaploidisation has had a profound impact on *Greyia* genome architecture, with approximately 40% of duplicated genomic regions retained, representing an unusually high level of preservation. Among the RDGs and RTGs, we observed significant enrichment for functions associated with photoperiod and circadian processes. Notably, palaeo‐hexaploidisation‐derived genes include key components of the photoperiod and circadian clock pathways involved in light perception (*CRY1*), core oscillation (*CCA1/LHY1*, *PRR5*, *PRR7*) and downstream regulation of flowering (*GI*, *EBS*). Their coordinated retention within palaeo‐hexaploidisation‐derived syntenic blocks suggests that polyploidy preserved an intact regulatory context rather than isolated gene copies. Photoperiodic regulation is a key mechanism by which plants align developmental transitions with seasonal environmental cues, particularly in temperate and subtropical regions. Although the present data do not establish a direct functional role for palaeo‐hexaploidisation‐derived photoperiod genes in shaping *Greyia* phenotypes, their retention patterns are consistent with a potential contribution to the regulation of seasonal growth habit between the evergreen *G. flanaganii* and the other two deciduous species. Tissue‐specific expression of these RTGs further indicate functional specialisation, implying that duplicated genes have been co‐opted for distinct roles in different organs or developmental stages. Preferential retention of circadian and photoperiod regulators following genome duplication has also been reported in other angiosperm lineages, supporting the idea that these pathways may be particularly dosage‐sensitive or constrained by network connectivity (Adams & Wendel, 2005; Soltis et al., 2015; Van de Peer et al., 2017, 2021). Together, these findings indicate that the *Greyia*‐specific palaeo‐hexaploidisation provided a genomic substrate upon which regulatory diversification could occur, with possible consequences for life‐history variation among species. Functional validation across developmental stages and seasonal contexts will be required to determine the extent to which retained photoperiod genes contribute to species‐specific traits in *Greyia*.

Trichome variation distinguishes *G. radlkoferi* with long non‐glandular trichomes (hairs) from the glabrous *G. sutherlandii* and *G. flanaganii*, yet their molecular bases have remained poorly understood. The overall conservation of candidate trichome‐development genes among the three species suggests that differences in gene content alone may not readily explain their contrasting trichome phenotypes. Instead, the expression patterns observed for several components of the trichome regulatory network raise the possibility that regulatory divergence has contributed to phenotypic differentiation. In Arabidopsis, *GL1* and *GL3/EGL3* are central components of the MYB–bHLH–WD40 complex, although the functions of these factors depend on their developmental context and interacting partners (Gonzalez et al., 2008; Nesi et al., 2001; Oppenheimer et al., 1991). The association between leaf‐enriched expression of particular *GL1* and *GL3/EGL3* homologues and the trichome phenotype of *G. radlkoferi*, together with the absence or altered tissue specificity of their expression in *G. sutherlandii*, is therefore compatible with regulatory differentiation of this pathway. Coordinated down‐regulation of upstream regulators, including *GIS3* and *GIS/GIS2*, further suggests attenuation of the trichome initiation module at the network level rather than isolated gene‐specific effects. Previous studies have demonstrated that promoter DNA methylation is generally associated with transcriptional repression in plants (Dubin et al., 2015; Zhang et al., 2018). Together with differential promoter methylation detected in *G. sutherlandii*, these patterns suggest regulatory divergence operating at both transcriptional and epigenetic levels. Moreover, polyploidisation has expanded the copy number of key MBW‐associated genes in *Greyia*, providing a substrate for differential expression and partitioning of regulatory roles among paralogs. However, copy retention and expression divergence alone do not demonstrate that polyploidisation directly drove trichome diversification. One copy may have retained the ancestral trichome‐related function, whereas other copies may have acquired divergent expression, weak residual expression, or functions unrelated to trichome development. Functional validation will therefore be required to determine whether specific copies retain ancestral roles or contribute to morphological divergence in *Greyia*.

In contrast to the pronounced regulatory divergence underlying leaf traits, the core floral organ identity gene network appears to be largely conserved among *Greyia* species. All three species share an identical complement of type II MADS‐box genes, including conserved ABCE‐class homologues with largely similar floral expression profiles, indicating strong conservation of the genetic framework specifying floral organ identity. However, because ABCE genes primarily function during early floral meristem establishment and organ specification, whereas our RNA‐seq data were obtained from flowers of mixed developmental stages rather than dissected early floral meristems or individual floral organs, the observed flower‐associated expression of candidate *AP1*, *AP3*, *PI*, *AG* and *SEP* homologues supports their annotation as floral organ development candidates in *Greyia*, but does not establish their specific developmental roles or link them directly to later floral shape traits. Notably, the retention of multiple *SEP* and *AG* homologues following the *Greyia*‐specific palaeo‐hexaploidisation provides an expanded regulatory repertoire that may have increased developmental flexibility during flower evolution. In rice, the *AG* homologous gene *OsMADS3* is essential for stamen identity and filament elongation, with *osmads3* mutants exhibiting shortened filaments and abnormal stamens (Castelan‐Munoz et al., 2019; Dreni et al., 2011). This finding raises the possibility that *AG* homologue expansion may have contributed to the evolution of the characteristic exserted stamens in *Greyia*. Likewise, the flower‐associated expression of *AGL6‐1*, particularly its elevated expression in *G. flanaganii*, further identifies this copy as a candidate for future studies of floral organ development in *Greyia*. Previous studies have shown that *AGL6*‐like genes can have diverse floral developmental roles across angiosperms: for example, *AGL6* is expressed in sepals and petals in water lily, whereas petunia and rice *AGL6*‐like genes show *SEP*‐like expression patterns (Rijpkema et al., 2009; Seok et al., 2010). However, these hypotheses remain speculative and will require developmental‐stage‐ and organ‐specific transcriptomic analyses together with functional validation.

Inflorescence development is governed by a complex network involving meristem maintenance, meristem identity, floral transition, branching, and hormone signalling. We surveyed candidate genes associated with these processes and examined *LFY* and *FT* in greater detail because promoter comparisons revealed their interspecific variation despite the broad conservation of their copy numbers and coding sequences. Such variation raises the possibility that regulatory changes, like we observed in trichome, rather than major alterations in protein‐coding regions, may contribute to differences in inflorescence development among *Greyia* species. In particular, the presence of predicted *AGL6* binding sites in the promoters of *G. radlkoferi* and *G. sutherlandii*, but not *G. flanaganii*, points to lineage‐specific modification of upstream regulatory context. In evergreen *G. flanaganii*, retention of leaves during flowering is compatible with a regulatory mode resembling canonical leaf‐derived *FT* signalling, whereas deciduous species that flower prior to leaf flush are more consistent with perennial tree strategies in which floral induction and initiation are temporally separated (Hsu et al., 2011; Wilkie et al., 2008). Interpretation of these patterns must consider the temporal constraints of transcriptomic sampling, as *FT* activity typically precedes anthesis and is restricted to narrow inductive windows. In this context, it is notable that the leaves of *G. radlkoferi* used for RNA‐seq were collected in late summer when the tree had not flowered for several years and did not flower again until spring of the next year, suggesting that the sampled tissues were unlikely to represent a floral inductive state. Thus, the absence of detectable *FT* expression in leaves of deciduous *Greyia* species is most parsimoniously explained by sampling context rather than by a lack of *FT* involvement in flowering regulation. The detection of *FT* transcripts in mature floral tissues of all three species is nevertheless intriguing and may reflect diversification of *FT* spatial or temporal roles, as reported for woody perennials (Wilkie et al., 2008). Overall, the observed promoter and expression differences identify *FT*, *LFY*, and their potential regulators as candidates for further investigation, but they do not establish that these genes cause the contrasting inflorescence phenotypes of *Greyia*. Other candidate genes without conspicuous differences in copy number, gene structure, or proximal promoter sequence may still contribute through changes in spatial or temporal expression, distal regulatory elements, epigenetic state, protein activity, or regulatory interactions. Dissecting the precise roles of *FT*, *LFY*, and *AGL6* during early floral induction versus later stages of floral development will require transcriptomic and spatial expression profiling of shoot apical meristems, combined with seasonal sampling across multiple developmental stages.

## CONCLUSION

This study presents chromosome‐scale genome assemblies for all three extant *Greyia* species, representing the first high‐quality genomic resources for Geraniales. Integrating comparative genomics with transcriptomic analyses, we show that polyploidy and regulatory diversification, rather than changes in gene content, underlie phenotypic variation in leaf trichomes as well as floral and inflorescence architecture. The retention of duplicated regulators following a *Greyia*‐specific palaeo‐hexaploidisation provided the genetic substrate for subfunctionalisation and regulatory reprogramming of conserved pathways. These high‐quality genomic resources establish a framework for functional dissection of developmental gene networks in Geraniales using *Greyia* as a model system. They can be deployed to elucidate the molecular basis of morphological differentiation in *Greyia*, for example adaptation to pollinators through floral architecture and nectary diversification (Jeiter, Weigend, & Hilger, 2017). Furthermore, they will provide the foundation for studies of adaptation to pests, pathogens, and abiotic stresses through chemical defences (Bohm & Chan, 1992), which will underpin exploitation of the medicinal properties of *Greyia* (Botha, De Canha, et al., 2026). Additionally, these genomic resources provide the resolution necessary to clarify the molecular systematics of *Greyia* and complement standard DNA barcoding approaches used for species identification (Botha, De Canha, et al., 2026). The establishment of reference genomes for each species facilitates robust population genomics across the genus's 1000 km range in southern Africa, offering a critical framework for evaluating species concepts in the face of recent divergence and the sympatric ranges of *G. radlkoferi* and *G. sutherlandii* (Botha, Maduna, et al., 2026). By securing the taxonomic identity of this keystone grassland genus, this would directly inform conservation strategies. On a macro‐evolutionary scale, these data will help resolve a long‐standing phytogeographic conundrum, namely that *Greyia* has closest phylogenetic affinity with *Francoa*, a genus endemic to Chile, and not other African taxa in Geraniales (*Melianthus*, *Bersama*) (Sytsma et al., 2014).

## EXPERIMENTAL PROCEDURES

### Plant materials and nucleic acid extraction

*Greyia* trees sampled in this study are part of a larger study where each individual tree was given a unique ‘P’ number. Plant samples for gDNA and RNA extraction were obtained from the following individual trees. *Greyia sutherlandii* Hook. & Harv. tree P245 growing at Monks Cowl Nature Reserve, Maloti‐Drakensberg Park World Heritage Site, KwaZulu‐Natal Province, South Africa. Sampling was conducted under Research Permits OP34/2022, OP737/2023, and OP2781/2024 issued by Ezemvelo KZN Wildlife, South Africa. *Greyia flanaganii* Bolus tree P261 at the Manie Van der Schijff Botanical Garden (accession number 4631), University of Pretoria, South Africa. *Greyia radlkoferi* Szyszyl. tree P262 at the Manie Van der Schijff Botanical Garden (accession number 4632), University of Pretoria, South Africa. *Greyia* species identities were determined based on flower and leaf morphological differences (Figure 1) verified against herbarium specimens at the HGWJ Schweickerdt Herbarium (PRU) at the University of Pretoria, South Africa. Representative voucher specimens of *G. sutherlandii* P245, *G. radlkoferi* P262 and *G. flanaganii* P261 have been deposited at this herbarium with accession numbers PRU0132900, PRU0133318, PRU0133319, respectively.

For high molecular weight DNA, fully expanded leaves at leaf flush stage were collected from *G. sutherlandii* P245, *G. flanaganii* P261, and *G. radlkoferi* P262, flash frozen in liquid nitrogen, and stored at −80°C. The protocol described by Zerpa‐Catanho et al. (2021) was followed to obtain high‐quality high molecular weight intact DNA: in short, nuclei were first isolated after thoroughly pulverising 5 g leaf material in liquid nitrogen using a nuclei isolation buffer (10 mM Tris–HCl, 10 mM EDTA, 100 mM KCl, 0.5 m sucrose, 4 mM spermidine, 1 mM spermine) to extract (supplemented with 10% Triton X‐102) and wash (supplemented with 0.1% β‐mercaptoethanol) the nuclei, from which DNA was extracted using a DNA extraction buffer (100 mM Tris–HCl, 50 mM EDTA, 500 mM NaCl, 1.25% SDS). The DNA was isolated from the extract by performing proteinase K and RNase A treatments, and using one volume phenol:chloroform:isoamyl alcohol (15:14:1) and chloroform:isoamyl alcohol (14:1) in subsequent steps to separate out cellular debris and other macromolecules. An equal volume of isopropanol was used to precipitate the DNA from solution, which was purified using a QIAGEN Genomic Tip 20/G (QIAGEN, Cat no./ID: 10 223). The DNA was resuspended in TE buffer and stored at 4°C prior to shipping to Macrogen Europe BV, Amsterdam for sequencing.

Material for RNA extraction was obtained by harvesting current season flower and/or leaf bearing branches of roughly 1.5 cm in diameter. To maintain tissue integrity during transit from the field sites, the cut ends of the branches were immediately placed in water and kept submerged until samples were taken for flash‐freezing. To match the genomic data, RNA was extracted once from pooled samples of each tissue type separately from the three individual *Greyia* trees selected for genome sequencing. Leaves selected for extraction were young and green, ranging from 2 to 5 cm in diameter and growing from new nodes at the tip of the branch. Flowers of various ages were harvested from inflorescences and pooled together for each extraction. This was to ensure a wider coverage of expressed genes for the annotation. Stem tissue (mostly made up of secondary phloem) for RNA extraction was recovered from 8‐cm segments of current‐season branches. A single longitudinal incision was made along each segment down to the central sapwood cylinder. The outer brown periderm layer was peeled off and discarded. The underlying green tissue layer, comprising the cortex and secondary phloem (approximately 2 mm thick), was then collected for RNA extraction by peeling it away at the vascular cambium layer from the sapwood and pith of the stem (Figure S16 for images of the material sampled).

RNA was extracted using a modified CTAB protocol with LiCl precipitation (Chang et al., 1993)—in short, 2 g of flash‐frozen tissue was ground to a fine powder in liquid nitrogen, to which pre‐warmed CTAB buffer (2 m NaCl, 25 mM EDTA, 100 mM Tris–HCl [pH 8.0], 2% PVP‐10, 2% CTAB, 3.44 mM spermidine, 2% β‐mercaptoethanol) was added. Samples were incubated for 10 min at 65°C before an equal volume of chloroform:isoamyl alcohol (14:1) was added and shaken vigorously. Centrifugation was performed at 8500 ***g*** at 4°C for 15 min, and the upper aqueous phase was transferred to a new, pre‐chilled Falcon tube. The chloroform:isoamyl alcohol extraction was repeated two more times. A ¼ volume 10 m LiCl was then added to precipitate the RNA overnight at 4°C, which was pelleted by centrifugation at 8500 ***g*** at 4°C for 90 min. Following the removal of the LiCl solution, the pellet was washed with ice‐cold 70% ethanol twice, with centrifugation performed at 8500 ***g*** at 4°C for 15 min. The pellet was air‐dried at room temperature and resuspended in RNase‐free water.

For DNase treatment, the RNA solution was first diluted to 0.2 μg μl^−1^ before 10× DNase I buffer (100 mM Tris [pH 7.5], 25 mM MgCl_2_, 5 mM CaCl_2_) and 2 U Ambion DNase I was added and incubated for 30 min at 37°C. The Monarch® Spin RNA Cleanup Kit (50 μg) (New England Biolabs, Ipswich, MA, USA; T2040) was then used to purify the RNA, and quality was assessed through Qubit and NanoDrop spectrophotometry (Thermo Fisher Scientific, Waltham, MA, USA). RNA was precipitated from solution with 0.1 volumes 3 m NaOAc (pH 5.5) and 2 volumes 100% ethanol for shipping on ice to Macrogen Europe BV, Amsterdam, The Netherlands.

### Genome sequencing

Long‐read nanopore sequencing was carried out on the PromethION sequencer (Oxford Nanopore Technologies, Oxford, UK) to assemble the genome. Genomic DNA by Ligation kit (SQK‐LSK110) was used to generate sequencing libraries following the manufacturer's protocol, and nanopore sequencing was performed on the R10.4.1 flow cells. Raw data were obtained from all PromethION runs and basecalling was performed with Dorado (v0.5.3).

Libraries were prepared for short‐read Illumina whole‐genome sequencing using the TruSeq® Nano DNA Library Prep Kit according to the protocol guide (Part # 15041110 Rev. D). The DNA was first fragmented to 550 bp fragments through Covaris shearing, leaving 3′ and 5′ overhangs, which were subsequently removed and repaired to produce blunt ends. The fragment 3′ ends are then adenylated to prevent ligation between fragments and provide a complementary overhang for the adaptors to anneal. Following ligation of the indexing adaptors, the fragments are selectively enriched through PCR amplification with primers complementary to the adaptors. The library concentrations are then normalised to 10 nM and pooled in equal volumes in the Pooled Diluted Cluster Template Plate for cluster generation. Paired‐end sequencing was performed using the Illumina NovaSeq platform of Macrogen Europe BV. The summary of ONT‐sequencing data is in Table S1a.

Hi‐C sequencing was conducted on the Illumina NovaSeq X plus system using the 150‐bp pair‐end reads by Biomarker Technologies (BMK) GmbH, Münster, Germany. The Hi‐C libraries were constructed to anchor scaffolds onto chromosomes (Burton et al., 2013). DNA isolated from *G. radlkoferi* P262 leaves was digested with HindIII overnight. Sticky ends were biotinylated and proximity‐ligated, and then physically sheared to a size of 300–700 bp and enriched to make chimeric junctions. The cross‐linked long‐distance physical connections were next processed into chimeric fragments, followed by reverse cross‐linking, purification, and PCR amplification, and were subsequently used to create paired‐end sequencing libraries.

For transcriptome sequencing, sequencing libraries were prepared using the TruSeq® Stranded mRNA LT Sample Prep Kit according to the protocol guide (#15031047 Rev. E)—in short, mRNA was first purified with oligo‐dT attached magnetic beads, fragmented, and used for cDNA synthesis. The 3′ ends of the cDNA were then adenylated to allow for adaptor ligation, following which the fragments are enriched through PCR amplification with primers specific to the adaptor sequences. Finally, the libraries are validated through quantification and quality control. The libraries were then loaded onto flow cells, with surface‐bound oligonucleotides capturing the complementary adaptors, and clonal clusters were produced from the captured fragments with bridge amplification. Paired‐end sequencing was performed using the Illumina NovaSeq platform of Macrogen Europe BV. The summary of RNA‐seq data is in Table S1b. Because RNA‐seq samples were not biologically replicated, expression data were used to support genome annotation and assess candidate gene expression across tissues, not for formal differential expression testing. The resulting expression patterns should therefore be interpreted as hypothesis‐generating and will require validation through replicated and spatially resolved expression analyses.

### Genome size and heterozygosity estimation

Genome size and heterozygosity were estimated by *k*‐mer frequency distribution analysis. Initially, the short reads were trimmed using Trimmomatic (v0.39) (Bolger et al., 2014) with the parameters ‘ILLUMINACLIP: TruSeq3‐PE‐2.fa:2:30:10 LEADING:20 TRAILING:20 SLIDINGWINDOW:10:20 MINLEN:50’. *K*‐mers were then counted using Jellyfish (v2.3.0) (Marcais & Kingsford, 2011) with the parameters ‘‐C ‐m 21 ‐s 4G ‐t 5’. The resulting output file was subsequently used as input for GenomeScope2.0 (Ranallo‐Benavidez et al., 2020), which estimated the genome size and heterozygosity using default parameters.

### Genome assembly and evaluation

Prior to the assembly, we performed quality trimming on the Nanopore data using Porechop (v0.2.4; https://github.com/rrwick/Porechop). Draft contig‐level assemblies were then generated with Flye (v2.9.3) using Nanopore data under optimised parameters (‐‐asm‐coverage 50; ‐‐nano‐hq).

Hi‐C sequencing reads were processed by removing adapters and filtering low‐quality sequences. These processed reads were then aligned to the contig‐level assembly using BWA (v0.7.17) (Li & Durbin, 2009), with the resulting BAM file serving as input for Juicer (v1.9.8) (Durand, Shamim, et al., 2016). Scaffolding was performed using 3D‐DNA (v180922) (Dudchenko et al., 2017) to cluster, order, and orient the contigs. Finally, we manually corrected misassembled scaffolds in Juicerbox (v1.11.08) (Durand, Robinson, et al., 2016) by examining Hi‐C contact frequency patterns. Genome completeness of the final assemblies was assessed using Benchmarking Universal Single‐Copy Orthologs (BUSCO, v5.7.1) (Manni et al., 2021) with the eudicots_odb10 dataset.

### Repeat prediction

A preliminary list of candidate LTR retrotransposons (LTR‐RT) was generated using LTR_FINDER_parallel (Ou & Jiang, 2019) and LTR_harvest (v1.5.9) (Ellinghaus et al., 2008). A repeats library was constructed for each *Greyia* species to include LTR‐RT and other TE elements identified by LTR_retriever (v3.0.1) (Ou & Jiang, 2018) and RepeatModeler (v2.0.1) (Flynn et al., 2020), respectively. The repeats library was used as input for RepeatMasker (v4.1.1; http://www.repeatmasker.org/RepeatMasker/) to predict and mask repeats in three *Greyia* genomes.

### Structural and functional annotation

Gene structure annotation in the three *Greyia* genomes was performed by integrating RNA‐based, homology‐based, and *ab initio* predictions. For RNA‐based prediction, RNA‐seq data were first mapped to the assemblies using HISAT2 (v2.1.0; parameter: –dta) (Kim et al., 2019), and subsequently assembled into transcripts by StringTie2 (v1.3.6) (Kovaka et al., 2019). All transcripts from RNA‐seq were combined using Cuffcompare (v.2.2.1) (Trapnell et al., 2010) and subsequently merged with Stringtie2 to remove fragments and redundant structures. TransDecoder (v5.0.2; https://github.com/TransDecoder/TransDecoder) was then used to predict open reading frames. *Arabidopsis thaliana* and *E. japonica* genomes and annotations were used for homology‐based prediction using GeMoMa (v1.9) (Keilwagen et al., 2016, 2018). BRAKER3 (v3.0.8) was used for *ab initio* prediction using model training based on RNA‐seq data.

All structural gene annotations were joined with EvidenceModeller (v1.1.1) (Haas et al., 2008). BUSCO (v5.7.1) and OMArk (v0.3.1) (Nevers et al., 2024) were used to assess the quality of the annotation results. Finally, we used GenomeView (Abeel et al., 2012) to perform the gene curation manually based on RNA‐seq data alignments, including (1) split incorrectly linked gene models, (2) added missing genes supported by RNA‐seq evidence, and (3) merged fragmented annotations where RNA‐seq indicated single transcripts. Putative gene functions were identified using InterProScan (v5.65) (Jones et al., 2014) and eggNOG‐mapper (v2.1.12) (Cantalapiedra et al., 2021).

### Whole‐genome duplication identification and dating

*K*_S_ age distribution analysis was performed using the wgd v2 package (Chen et al., 2024). Anchor pairs (i.e., paralogous genes lying in collinear or syntenic regions of the genome) were identified using i‐ADHoRe (Proost et al., 2012). To account for substitution rate heterogeneity among lineages, *K*_S_ distribution analysis among species was also corrected using the KSRATES software (Sensalari et al., 2022), which locates ancient polyploidisation events with respect to speciation events within a phylogeny, comparing paralog and orthologue *K*_S_ distributions while correcting for substitution rate differences across the involved lineages. Visualisation of the *K*_S_ distribution was performed with Hiplot (https://hiplot.cn/).

The absolute age (in geological time) of the palaeo‐hexaploidisation event in *Greyia* and the WGD event in Euscaphis was also dated using the wgd v2 package (Chen et al., 2024). In brief, paralogous gene pairs located in duplicated segments (so‐called anchor genes, anchor pairs, or anchors) and duplicated pairs lying under the WGD peak (so‐called peak‐based duplicates) were collected for phylogenetic dating. Anchors, assumed to be corresponding to the most recent WGD, were detected using i‐ADHoRe (Fostier et al., 2011; Proost et al., 2012; v3.0). We selected anchor pairs and peak‐based duplicates present under the *Greyia* WGD peak and with *K*_S_ values between 0.3 and 0.8 for absolute dating. For each supposed WGD paralogous pair, an orthogroup was created that included the two paralogs plus several orthologues from other plant species as identified by InParanoid (Ostlund et al., 2010; v4.1) using a broad taxonomic sampling: *Lotus japonicus*, *Vigna mungo*, *Tripterygium wilfordii*, *Poncirus trifoliata*, *Mangifera indica*, *Pistacia vera*, *Theobroma cacao*, *Durio zibethinus*, *Gossypium raimondii*, *A. thaliana*, *Tarenaya hassleriana*, *Cleome violacea*, *G. radlkoferi*, *Paeonia ostii* and *Kalanchoe fedtschenkoi*. In total, 780 orthogroups based on anchors and peak‐based duplicates were collected for the phylogenetic dating. The pipeline can be divided into three main steps. (1) The construction of anchor *K*_S_ distribution and the delineation of credible *K*_S_ range adopted for phylogenetic dating, using ‘wgd dmd’, ‘wgd ksd’, ‘wgd syn’ and ‘wgd peak’. (2) The formulation of a starting tree used in the phylogenetic dating, composed of a broad taxonomic species with annotated fossil calibration information, using ‘wgd dmd ‐‐globalmrbh’. (3) The construction of orthogroups consisting of collinear duplicates of *Greyia* species and their reciprocal best hits (RBHs) against other species in the starting tree and the phylogenetic dating using a molecular dating program MCMCtree with the parameters ‘burnin = 2000 ‐ds sampfreq = 1000' ‐ds nsample = 20000’ via wgd focus. Finally, 780 orthogroups were accepted, and absolute age estimates of the node uniting the WGD paralogous pairs based on both anchor pairs and peak‐based duplicates were grouped into one absolute age distribution, for which kernel density estimation and a bootstrapping procedure were used to find the peak consensus WGD age estimate and its 90% CI boundaries, respectively.

### Phylogenetic tree construction and estimation of divergence times

To reconstruct the evolutionary history of Geraniales, we sampled representative genomes from major rosid lineages, including Brassicales (*A. thaliana*), Malvales (*T. cacao*), Sapindales (*Citrus clementina*), Oxalidales (*Averrhoa carambola*), Malpighiales (*Euphorbia peplus*), Celastrales (*T. wilfordii*), Fagales (*Castanea dentata*), Rosales (*Prunus persica*), Cucurbitales (*Cucumis sativus*), Fabales (*L. japonicus*), Geraniales (*G. flanaganii*, *G. radlkoferi* and *G. sutherlandii*), Crossosomatales (*E. japonica*), Zygophyllales (*Zygophyllum xanthoxylum*) and Myrtales (*Eucalyptus grandis* and *Punica granatum*) (Table S2). Two monocots, *Oryza sativa* and *Brachypodium distachyon*, were included as outgroups. Orthologous groups (https://bioinformatics.psb.ugent.be/gdb/Greyia/Orthogroups.tsv) were identified using OrthoFinder (v2.5) (Emms & Kelly, 2019). From these results, single‐copy orthologous genes were extracted. Protein sequences of each single‐copy orthologue were aligned with MAFFT (Katoh & Standley, 2013) using default parameters, and the resulting protein alignments were converted into codon alignments. Poorly aligned or divergent regions were removed using trimAl (Capella‐Gutierrez et al., 2009). The filtered codon alignments from all single‐copy orthologues were then concatenated into a supergene matrix for species phylogenetic reconstruction. A maximum‐likelihood phylogenetic tree was inferred from both the protein and codon alignments using IQ‐TREE v2.1.2 (Nguyen et al., 2015) under the GTR+G and TEST model with 1000 bootstrap replicates. Coalescent‐based species trees were also reconstructed independently from the nucleotide and amino acid datasets. Each gene tree was independently inferred for each locus using IQ‐TREE v2.1.2. The best‐fitting substitution model for each alignment was selected using the TEST model parameter and branch support was evaluated using 1000 ultrafast bootstraps. The resulting maximum‐likelihood gene trees were used to estimate a species tree under the multispecies coalescent model in ASTRAL v5.7.1 (Mirarab et al., 2014). To reduce the influence of poorly resolved gene‐tree branches, branches with ultrafast bootstrap support below 10% were collapsed before species‐tree estimation. Branch support in the ASTRAL tree was evaluated using local posterior probabilities. The resulting species trees were rooted using *B. distachyon* and *O. sativa* as outgroups. Gene‐tree discordance was evaluated using the normalised quartet frequencies calculated by ASTRAL v5.7.1. The nucleotide‐ and amino‐acid‐based gene trees were analysed separately. Gene trees from the 401 single‐copy loci were used as input. We focused on the three alternative relationships among malvids, fabids, and the Geraniales‐Crossosomatales lineage (*Greyia* + *Euscaphis*). The three normalised quartet frequencies were assigned to the corresponding rooted topologies: q1, ([Malvids, Fabids], *Euscaphis* + *Greyia*); q2, ([Fabids, *Euscaphis* + *Greyia*], Malvids); and q3, ([*Euscaphis* + *Greyia*, Malvids], Fabids). These values quantify the relative frequencies of induced gene‐tree quartets supporting each alternative and sum to one.

Divergence times among 19 plant species were estimated with MCMCtree in the PAML package (Yang, 2007), with the concatenated topology specified as the phylogenetic framework, employing the GTR+G substitution model. Fossil‐based calibration points included: the divergence between monocots and eudicots (125–247 Mya; based on tricolpate pollen grain) (Dilcher et al., 2007), the split between *B. distachyon* and *O. sativa* (42–52 Mya) (Catalan et al., 2012), and the divergence between fabids and malvids (102–125 Mya) (Wang et al., 2009). For Bayesian inference, MCMCtree was run to generate 10 000 posterior trees, sampling every 150 iterations after a burn‐in of 500 000 iterations. Two independent runs were compared to assess convergence, and the posterior distribution of node ages was also compared with the effective prior implied by fossil calibrations.

To place *Greyia* within Geraniales using broader taxon sampling, translated transcriptomes from six Geraniales species with BUSCO completeness >60% (Table S3) were combined with predicted protein sets from the three *Greyia* genomes. Orthogroups were inferred using OrthoFinder (v2.5), and the species tree was reconstructed from low‐copy genes generated by the OrthoFinder.

### Genome and gene family evolution

Synteny analyses were conducted using MCScanX with the parameter ‘‐s 10.’ Genes duplicated or triplicated as a result of palaeo‐hexaploidisation events were visualised with Circos (Krzywinski et al., 2009). Syntenic relationships among *Euscaphis* and *Greyia* were further identified using JCVI (https://github.com/tanghaibao/jcvi). Gene Ontology (GO) enrichment analyses were performed with the R package clusterProfiler (Wu et al., 2021).

For expression analysis, RNA‐seq data were first mapped to the assemblies using HISAT2 (v2.1.0; parameter: –dta) and then raw reads counts were obtained using featureCounts (v2.0.1) (Liao et al., 2014), TPM values were calculated with a custom script (https://github.com/JiyangChang/XiaoTools/blob/main/Misc/TPM_calculator.py).

For MADS‐box gene identification, a hidden Markov model (HMM) approach was applied to the three *Greyia* genomes. The HMM profile for the SRF‐TF domain (PF00319) was retrieved from the Pfam database (Mistry et al., 2021). Candidate MADS‐box genes were identified by searching the local protein databases with HMMER (Eddy, 2011) using an *E*‐value cutoff of <1e‐5. MADS‐box proteins of *A. thaliana* were identified using the same pipeline. Candidate sequences from all four species were then aligned with MAFFT and concatenated for phylogenetic reconstruction, and the resulting gene tree was visualised with iTOL (https://itol.embl.de/login.cgi). Trichome‐related genes in *Greyia* were identified via homology searches against *A. thaliana*. RNA expression profiles of MADS‐box and trichome‐related genes were visualised using Chiplot (https://www.chiplot.online/).

For promoter analysis, promoter regions were operationally defined as the 2‐kb sequence upstream of the translation start site (ATG) (or upstream of the annotated TSS when available) for *cis*‐element/TFBS scanning, following common practice in plant promoter studies (Baek et al., 2011; Brooks et al., 2023; Thibaud‐Nissen et al., 2006). Thus, the 2‐kb sequence upstream of each target gene was extracted as the putative promoter region. Motif discovery was performed with MEME (http://meme‐suite.org) to identify statistically overrepresented sequence patterns. The predicted motifs were subsequently annotated and compared against TF binding site databases using Tomtom (https://meme‐suite.org/meme/tools/tomtom), thereby enabling functional inference of *cis*‐regulatory elements.

For DNA methylation analysis, Oxford Nanopore reads for genome assembly were basecalled using Dorado with modified base detection enabled (‐‐modified‐bases‐models), allowing the identification of methylated bases directly from raw signal data. Basecalled reads were aligned to the reference genome using the Dorado aligner, and methylation calls were extracted from the resulting BAM files using modkit and converted to BED format for downstream analyses.

### Microscopy

Abaxial and adaxial surfaces of young leaves (~2 cm in diameter) were visualised using a Keyence VHX‐7000 digital microscope (Keyence Corporation of America, Itasca, IL, USA). Images were captured under 150× amplification and fine depth composition was used to improve the focus of the three‐dimensional surface.

Leaf cross section images were prepared by fixing leaves of a similar age to those used for the digital microscopy in formalin acetic acid alcohol (FAA) for a few days. Histological methods for Safranin O and Fast Green staining were adapted from Johansen (1940) and Criswell et al. (2025). The fixed tissue was dehydrated using an ethanol concentration series, and stained with Safranin O for visibility during sectioning. The tissue was then treated with a graded ethanol:xylene series to impregnate the tissue with xylene, which is miscible with paraffin wax but not water, to allow for wax infiltration. The xylene was gradually saturated with paraffin wax and then replaced in fractions with liquid paraffin, until the remaining xylene was eventually allowed to evaporate. The wax‐infiltrated tissue was mounted in clean paraffin wax and sectioned at a thickness of 5 μm with a microtome. The wax ribbons were placed on microscope slides covered with a thin layer of Haupt's gelatin adhesive and incubated overnight at 60°C to melt the wax out of the tissue, and subsequently exposed to formalin fumes for 8 h to activate the adhesive prior to staining.

Wax was removed from the slide by washing in xylol for 2 min followed by 50% ethanol. The slides were stained with Safranin O for 15 min and washed with 70 and 90% ethanol, and subsequently counterstained with Fast Green for 35 sec. The slides were then washed with 100% ethanol, ethanol:xylene (1:1 v/v), and finally xylene to remove excess stain. After airdrying, a few drops of Entellan™ mounting liquid were applied and the cover slip placed in position. Slides were viewed at 400× magnification using a Nikon Eclipse E200 compound microscope.

## AUTHOR CONTRIBUTIONS

XM: data curation, formal analysis, software, visualisation, writing—original draft (lead), methodology (equal). JC: data curation, formal analysis, software, visualisation (lead), methodology, writing—review and editing (equal). JB: investigation (lead), methodology, visualisation, writing—original draft (supporting). YVdP: resources, funding acquisition (lead), conceptualisation, project administration, supervision, writing—review and editing (equal). DKB: conceptualisation, project administration, supervision, writing—review and editing (equal), resources, funding acquisition (supporting).

## CONFLICT OF INTEREST

The authors have no conflict of interest to declare.

## Supporting information


**Figure S1.** Genome size and heterozygosity of *G. radlkoferi* (left) and *G. flanaganii* (right) based on 21‐mer analysis using Illumina short reads.
**Figure S2.** The circos figures of *G. sutherlandii* (left panel) and *G. flanaganii* (right panel) generated using the *G. radlkoferi* genome as the reference for genome‐guided scaffolding.
**Figure S3.** Concatenated‐ and ASTRAL‐based phylogenetic trees.
**Figure S4.** The phylogenetic relationships within Geraniales using *Arabidopsis thaliana* and *Theobroma cacao* as the outgroup and including *Euscaphis japonica*.
**Figure S5.** Absolute dating (see Methods) of the *E. japonica* WGD event.
**Figure S6.** The macrosynteny among *G. radlkoferi*, *G. sutherlandii* and *G. flanaganii* using MCScanX.
**Figure S7.** The micro‐synteny of three inversions found among *G. radlkoferi*, *G. sutherlandii*, and *G. flanaganii* using MCScanX.
**Figure S8.** The GO enrichment (*P* < 0.05) of duplicated gene families retained from the WGT event in *Greyia*.
**Figure S9.** The GO enrichment (*P* < 0.05) of triplicated gene families retained from the WGT event in *Greyia*.
**Figure S10.** The expression profile of duplicated and triplicated genes involved in the photoperiodic module of *G. radlkoferi*.
**Figure S11.** 13 MYB transcription factors with significantly higher expression in leaves than in flowers and stem tissues of *G. radlkoferi*, together with their homologues in *G. sutherlandii*.
**Figure S12.** The gene structures and domain regions of *TFL1*, *LFY* and *FT* in the three *Greyia* species.
**Figure S13.** A 226‐bp insertion was identified in the *LFY* promoter of *G. flanaganii*.
**Figure S14.** Comparison of *FT* expression levels in flower and leaf of *G. radlkoferi*, *G. sutherlandii* and *G. flanaganii*.
**Figure S15.** Comparison of *AGL6* expression levels in flower and leaf of *G. radlkoferi*, *G. sutherlandii* and *G. flanaganii*.
**Figure S16.** Preparation of *Greyia* stem tissue used for RNA extraction.

**Table S1a.** The summary of ONT‐sequencing data.
**Table S1b.** The summary of RNA‐sequencing data.
**Table S1c.** Mapping rates (%) of RNA‐seq reads from multiple tissues.
**Table S2.** Plant genomes used in the phylogenomic analyses.
**Table S3.** Transcriptomic data for species within Geraniales.
**Table S4.** Retention of duplicated genes in three *Greyia* spp.
**Table S5.** Methylated sites detected in the promoter of *GL1* in *G. sutherlandii*.
**Table S6.** Methylated sites detected in the promoter of *GIS3* in *G. sutherlandii*.
**Table S7a.** Type II MADS‐box genes and encoded proteins.
**Table S7b.** Count of Type II MADS‐box genes and encoded proteins.
**Table S8.** Predicted TFBSs across the 2‐kb upstream promoter region of *LFY* in *G. flanaganii* (A of ATG located at position 2000).
**Table S9.** Predicted TFBSs across the 1.77‐kb upstream promoter region of *LFY* in *G. radlkoferi* (A of ATG located at position 1772).
**Table S10.** Predicted TFBSs across the 1.77‐kb upstream promoter region of *LFY* in *G. sutherlandii* (A of ATG located at position 1769).
**Table S11.** Predicted TFBSs across the 2‐kb upstream promoter region of *FT* in *G. flanaganii* (A of ATG located at position 2000).
**Table S12.** Predicted TFBSs across the 2‐kb upstream promoter region of *FT* in *G. radlkoferi* (A of ATG located at position 2000).
**Table S13.** Predicted TFBSs across the 2‐kb upstream promoter region of *FT* in *G. sutherlandii* (A of ATG located at position 2000).

## Data Availability

Processed RNA‐seq data, genome assemblies, structural and functional annotations, and protein‐coding genes are deposited in the ORCAE database. *Greyia flanaganii* is available: https://bioinformatics.psb.ugent.be/gdb/Greyia/Greyia_flanaganii/. *Greyia radlkoferi* is available: https://bioinformatics.psb.ugent.be/gdb/Greyia/Greyia_radlkoferi/. *Greyia sutherlandii* is available: https://bioinformatics.psb.ugent.be/gdb/Greyia/Greyia_sutherlandii/. The ONT Nanopore genome sequencing data of three Greyia are available https://bioinformatics.psb.ugent.be/gdb/Greyia/nanopore_data/. The ONT Nanopore genome sequencing data for the three *Greyia* species, together with the raw RNA‐seq data from leaf, flower and stem tissues, have also been deposited in NCBI under BioProject PRJNA1417624 (https://www.ncbi.nlm.nih.gov/sra/?term=PRJNA1417624).
