# Supplementary material for: Genomic insights into trichome and inflorescence evolution of the palaeopolyploid tree genus Greyia
Source: Plant J. 2026 Oct 20;128(1):e71134. doi: 10.1111/tpj.71134 (PMC13680974; doi:10.1111/tpj.71134)
Supplement: Supplementary file 1 [file TPJ-128-0-s002.docx]

**Supplementary Figures**

**Genomic insights into trichome and inflorescence evolution of the palaeopolyploid tree genus *Greyia***

Xiao Ma^1,2^, Jiyang Chang^1,2^, Jana Botes^3^, Yves Van de Peer^1,2,4,5*^, Dave Kenneth Berger^3*^

1. Department of Plant Biotechnology and Bioinformatics, Ghent University, 9052 Ghent, Belgium
2. VIB Center for Plant Systems Biology, 9052 Ghent, Belgium
3. Department of Plant and Soil Sciences, Forestry and Agricultural Biotechnology Institute (FABI), University of Pretoria, Pretoria, 0028 South Africa
4. Department of Biochemistry, Genetics and Microbiology, University of Pretoria, Pretoria, 0028 South Africa
5. College of Horticulture, Academy for Advanced Interdisciplinary Studies, Nanjing Agricultural University, Nanjing, 210095 China
6. *	Corresponding authors

**Figure S1.** Genome size and heterozygosity of *G. radlkoferi* (left) and *G. flanaganii* (right) based on 21-mer analysis using Illumina short reads.

**Figure S2.** The circos figures of *G. sutherlandii* (left panel) and *G. flanaganii* (right panel) generated using the *G. radlkoferi* genome as the reference for genome-guided scaffolding. The tracks from the inside to the outside correspond to Copia elements, Gypsy elements, gene density per 100 kb, and chromosomes. The innermost grey curves represent all syntenic regions.

**Figure S3.** Concatenated- and ASTRAL-based phylogenetic trees. a. Phylogenetic trees based on the concatenated (left) and multi-species coalescent methods (right) based on 401 single-copy genes. b. Estimated proportions of the single-copy gene trees with different topologies based on nucleotide and amino acid sequence alignments. The x-axis labels q1, q2, and q3 refer to the quartet support for the first alternative, the main topology, and the second alternative, respectively.

**Figure S4.** The phylogenetic relationships within Geraniales using *Arabidopsis thaliana* and *Theobroma cacao* as the outgroup and including *Euscaphis japonica*. The tree was reconstructed from 2,479 orthogroups, each containing single-copy genes in at least seven of the twelve sampled species. Species in Geraniales are annotated in the green box. The blue and red lines indicate two major Geraniales clades: one comprising *Pelargonium dichondrifolium, Monsonia marlothii, California macrophylla, and Erodium chrysanthum*; and the other including *Greyia, Melianthus villosus, and Francoa sonchifolia*. The number at internal nodes represents STAG (Species Tree of All Genes) support percentages. Transcriptome datasets are listed in Table S3. The datasets of *A. thaliana*, *T. cacao* and *E. japonica* are listed in Table S2.

**Figure S5.** Absolute dating (see Methods) of the *E. japonica* WGD event. The age distribution was obtained by phylogenomic dating of *E. japonica* paralogs. The solid black line represents the Kernel Density Estimation (KDE) of the dated paralogs, while the vertical solid black line represents its peak at 76.18 MYA, which was used as the consensus WGD age estimate. The vertical black dotted lines represent the corresponding 90% confidence interval (CI) for the WGD age estimate, 48.99-99.66 MYA. The green histogram shows the raw distribution of dated paralogs.

**Figure S6.** The macrosynteny among *G. radlkoferi*, *G. sutherlandii*, and *G. flanaganii* using MCScanX. The vertical green lines represent the inversions.

**Figure S7.** The micro-synteny of three inversions found among *G. radlkoferi*, *G. sutherlandii,* and *G. flanaganii* using MCScanX.

**Figure S8.** The GO enrichment (p<0.05) of duplicated gene families retained from the WGT event in *Greyia*. The left panel showed that 2518 families are common in three *Greyia* species. The right panel is GO enrichment of shared WGT-derived duplicated genes in *Greyia* (2518 gene families).

**Figure S9.** The GO enrichment (p<0.05) of triplicated gene families retained from the WGT event in *Greyia*. The left panel showed that 1080 genes (360 triple-copy paleoparalogs) consistently retained across all *Greyia* species belonged to 336 families. The right panel is GO enrichment of shared WGT-derived triplicated genes (three copies) in *Greyia* (336 gene families).

**Figure S10.** The expression profile of duplicated and triplicated genes involved in the photoperiodic module of *G. radlkoferi*.

**Figure S11.** 13 MYB transcription factors with significantly higher expression in leaves than in flowers and stem tissues of *G. radlkoferi,* together with their homologs in *G. sutherlandii*. Each row represents the homologous genes in two *Greyia* species. Among these genes, Grera0712050 with leaf-specific expression and Gresu07g12200 with no detectable expression across all tissues are the homologs of *GL1* in *G. radlkoferi* and *G. sutherlandii,* respectively.

**Figure S12**. The gene structures and domain regions of *TFL1*, *LFY*, and *FT* in the three *Greyia* species.

**Figure S13.** A 226-bp insertion was identified in the *LFY* promoter of *G. flanaganii*. Multiple sequence alignment of the 2-kb upstream regions from the transcription start site (TSS) revealed that this insertion is located between 1335 and 1660 bp in the alignment, corresponding to 341–566 bp upstream of the TSS. This insertion is absent in *G. radlkoferi* and *G. sutherlandii*.

**Figure S14.** Comparison of *FT* expression levels in flower and leaf of *G. radlkoferi*, *G. sutherlandii*, and *G. flanaganii*.

**Figure S15.** Comparison of *AGL6* expression levels in flower and leaf of *G. radlkoferi*, *G. sutherlandii*, and *G. flanaganii*.

**Figure S16**. Preparation of *Greyia* stem tissue used for RNA extraction. The outer phellem consists of dead cells. This could easily be removed and discarded from the underlying green cortex owing to a layer of meristematic cells between the phellum and cortex. The green cortex and secondary phloem layer could then also be peeled away from the inner xylem and pith tissues through separation at the vascular cambium layer. As the cortex and secondary phloem layer consisted mainly of living cells, this layer was retained and used for RNA extraction. The panel on the right-hand side is a light microscopy image with scale bar = 0.5 µm (Image credit: AE Van Wyk).
